# Bioassay-Guided Isolation and Identification of Cytotoxic Compounds from *Gymnosperma glutinosum* Leaves

**DOI:** 10.3390/molecules170911229

**Published:** 2012-09-20

**Authors:** Ramiro Quintanilla-Licea, Rolando Morado-Castillo, Ricardo Gomez-Flores, Hartmut Laatsch, María Julia Verde-Star, Humberto Hernández-Martínez, Patricia Tamez-Guerra, Reyes Tamez-Guerra, Cristina Rodríguez-Padilla

**Affiliations:** 1 Departamento de Química, Facultad de Ciencias Biológicas, Universidad Autónoma de Nuevo León, Av. Universidad S/N, Cd. Universitaria, San Nicolás de los Garza 66451, Nuevo León, Mexico; 2 Departamento de Microbiología e Inmunología, Facultad de Ciencias Biológicas, Universidad Autónoma de Nuevo León, Av. Universidad S/N, Cd. Universitaria, San Nicolás de los Garza 66451, Nuevo León, Mexico; 3 Institute of Organic and Biomolecular Chemistry, Georg-August-University of Göttingen, Tammannstrasse 2, Göttingen D-37077, Germany

**Keywords:** *Gymnosperma glutinosum*, asteraceae, Mexican medicinal plant, lymphoma cells, cancer, *ent*-Labdanediterpenes

## Abstract

Bioassay-guided fractionation of hexane extracts of *Gymnosperma glutinosum* (Asteraceae) leaves, collected in North Mexico, afforded the known compounds hentriacontane (**1**) and (+)-13*S*,14*R*,15-trihydroxy-*ent*-labd-7-ene (**2**), as well as the new *ent*-labdane diterpene (−)-13*S*,14*R*,15-trihydroxy-7-oxo-*ent*-labd-8(9)-ene (**3**). In addition, D-glycero-D-galactoheptitol (**4**) was isolated from the methanolic extract of this plant. Their structures were established on the basis of high-field 1D- and 2D NMR methods supported by HR-MS data. The cytotoxic activity was determined by using the *in vitro* L5178Y-R lymphoma murine model. Hentriacontane (**1**) and the new *ent*-labdane **3** showed weak cytotoxicity, whereas the *ent*-labdane **2** showed significant (*p* < 0.05) and concentration dependent cytotoxicity (up to 78%) against L5178Y-R cells at concentrations ranging from 7.8 to 250 µg/mL.

## 1. Introduction

Cancer is still the second leading cause of death in industrialized countries [1]. The fate of many cancer patients, for whom cure of their disease is not a reality, is becoming ever more an issue. As repeatedly shown by the National Cancer Institute (USA), more than two thirds of the anticancer drugs approved between the 1940s and 2006 are either natural products or were developed based on the knowledge gained from natural products [2,3,4,5]. 

Phytotherapy represents the oldest form of therapy worldwide; more than 21,000 plant species are used in herbal medicines, according to the World Health Organization [1]. In particular, phytotherapy is practiced by a large part of the Mexican population for the treatment of many diseases. To promote the proper use of herbal medicines and to determine their potential use as source for new drugs, it is essential to study medicinal plants and scientifically validate their use [6,7,8,9]. This is the case for *Gymnosperma* *glutinosum* (Spreng.) Less, (Asteraceae) variously known in Mexico as *Tatalencho*, *Jarilla*, *Mota*, *Hierba Pegajosa*, *Escobilla* and *Pegajosa* [10], which is traditionally used to treat diarrhea, ulcers and rheumatism [11]. This shrubby, monotypic plant is distributed from Guatemala throughout Mexico to the southwestern region of the United States [12,13]. It is closely related to the *Gutierrezia* genus, small shrubby plants with slender stems that are herbaceous above and woody near the base, covered from summer through fall with masses of tiny yellow flowers borne in terminal clusters. The stems, leaves and flowering heads are covered with a glutinous or sticky substance.

**Figure 1:**
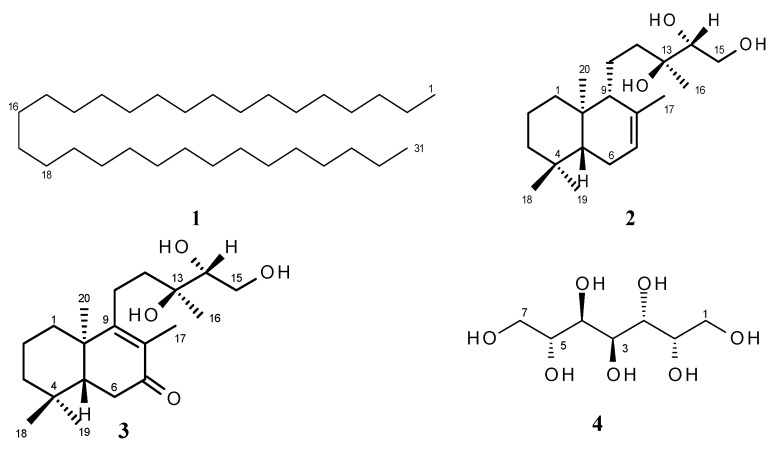
Structures of compounds **1**–**4**.

It has been reported that *Gymnosperma glutinosum* contains essential oils [14], flavonoids [15,16,17,18,19] and diterpenes [20,21,22,23,24], and recent studies have validated the ethnobotanical use of this plant in Mexico [24,25,26]. Gomez-Flores *et al.* showed anti-tumor activity of the hexane extract of *G. glutinosum *leaves against L5178Y-R lymphoma cells [27]; we have expanded our investigation on this plant by elucidating the chemical structure of the putative bioactive compounds present in *G. glutinosum*. We describe now the bioassay-guided isolation of hentriacontane (**1**), (+)-13*S*,14*R*,15-trihydroxy-*ent*-labd-7-ene (**2**) and (−)-13*S*,14*R*,15-trihydroxy-7-oxo-*ent*-labd-8(9)-ene (**3**), a new *ent*-labdane diterpene, as well as D-glycero-D-galactoheptitol (**4**). The structure of these compounds (Figure 1) was elucidated by analysis of the spectroscopic data and their cytotoxic activity was determined by using the *in vitro* L5178Y-R lymphoma murine model [27].

## 2. Results and Discussion

### 2.1. Structure Elucidation

The structures of the known compounds **1**, **2** and **4** were established by comparison of their spectroscopic data with literature values. Hentriacontane (**1**), a solid long-chain alkane (C_31_H_64_), is found in a variety of plants [28,29], but it has not yet been isolated from *G. glutinosum*. The occurrence of the e*nt*-labdane diterpene **2** (C_20_H_36_O_3_) in *G. glutinosum* has been reported in the literature [22], but no biological activity was described so far.

Compound **3** was isolated as a colorless oil with [α]_D_^20^ = −16.5 (c 2.0, CHCl_3_). (+)-ESI MS exhibited an ion peak [M+Na]^+^ at *m/z* = 361 (C_20_H_34_O_4_Na) and HR-ESI-MS showed an [M+H]^+^ ion at *m/z* = 339.25300, confirming a molecular formula of C_20_H_34_O_4_. The ^1^H-NMR spectrum (Table 1) showed five methyl singlets at δ_H_ = 0.85, 0.87, 1.05, 1.22 and 1.72, characteristic signals of a labdane diterpene skeleton [30,31,32]. The ^13^C-NMR spectrum of **3** (Table 1), in agreement with the molecular formula, revealed signals corresponding to 20 carbon atoms. Analysis of the ^13^C-NMR and DEPT 135 spectral data with the aid of the HSQC spectra, led to the deduction of the multiplicities of the carbon atoms and established the presence of five methyl signals (11.3, 18.1, 21.3, 22.7, 32.5), two tetrasubstituted olefinic carbons (130.1, 168.6), seven aliphatic methylenes (18.6, 23.1, 35.1, 35.9, 36.4, 41.2, 63.2), two methines (50.2, 70.2), one oxygenated quaternary carbon (74.4), one carbonyl group (200.5), and two quaternary carbons (33.1, 41.1). The above signals and an ABX system in the ^1^H-NMR spectrum of **3** for protons geminal to hydroxyl functions (one methylene (δ_H_ = 3.69, m, 2H; δ_C_ = 63.2) and one methine (δ_H_ = 3.49, m, 1H; δ_C_ = 77.2) corresponding to a primary and secondary alcohol) indicated a diterpene with *ent*-labdane skeleton whose structure is similar to the (+)-13*S*,14*R*,15-trihydroxy-*ent*-labd-7-ene (**2**) previously isolated by Maldonado from *Gymnosperma glutinosum* [22]. 

**Table 1:** ^1^H (400 MHz) and ^13^C-NMR (100 MHz) data of compound **3** (CDCl_3_).

Position	^13^C		^1^H
	δ	(DEPT)	δ,mult,(*J *in Hz)
1	35.9	CH_2_	1.27 m, 1.85 m
2	18.6	CH_2_	1.45 m, 1.54 m
3	41.2	CH_2_	1.16 m, 1.40 m
4	33.1	C_q_	-
5	50.2	CH	1.67 m
6	35.1	CH_2_	2.31 m, 2.41 m
7	200.5	C=O	-
8	130.1	C_q_	-
9	168.6	C_q_	-
10	41.1	C_q_	-
11	23.1	CH_2_	2.22 m, 2.36 m
12	36.4	CH_2_	1.43 m, 1.64 m
13	74.4	C_q_	-
14	77.2	CH	3.49 m
15	63.2	CH_2_	3.69 m
16	22.7	CH_3_	1.22 s
17	11.3	CH_3_	1.72 s
18	32.5	CH_3_	0.85 s
19	21.3	CH_3_	0.87 s
20	18.1	CH_3_	1.05 s
OH			3.60 br s

**Figure 2:**
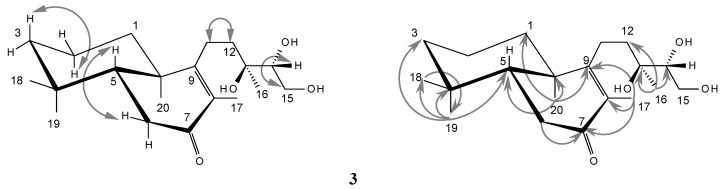
Selected H,H COSY (↔) and HMBC (→) correlations of diterpene **3** in CDCl_3_.

Furthermore, comparison of the ^1^H- and ^13^C-NMR spectral data of **3** with **2** allowed the assignment of **3** as (−)-13*S*,14*R*,15-trihydroxy-7-oxo-*ent*-labd-8(9)-ene, as compound **3** did not show any olefinic proton signal in the ^1^H-NMR spectrum (δ_H_ = 5.37 in **2**), and in the ^13^C-NMR spectrum only two signals for methine carbons (three methine signals in **2**) were present, but additionally a ketocarbonyl signal (δ_C_ = 200.5) was found. An ABX signal at δ_H_ 2.31/2.41, not present in the spectrum of **2**, showed an HMBC coupling with the carbonyl group (δ_C_ = 200.5) and a COSY correlation with H-5 (δ_H_ = 1.67). It corresponds therefore to CH_2_-6, indicating a change of the double bond in **2** from position Δ^7^ to position Δ^8^ in **3** as well as the presence of a carbonyl function at carbon 7 in **3**. This was confirmed by the HMBC correlation of Me-17 with CO-7 (and C-8, 9) and by the coupling of Me-20 with the β-olefinic carbon C-9. Further HMBC couplings are summarized in Figure 2. 

The IR spectrum of **3** displayed accordingly absorption bands at 1640, 1690 and 3370 cm^−1^, corresponding to a double bond, an α,β-unsaturated keto group and hydroxyl functions. We assume in **3** the same configuration as in **2** for the respective chiral carbons (5, 10, 13 and 14), as **3** is probably formed by oxidation of **2** in the plant cells.

**Figure 3:**
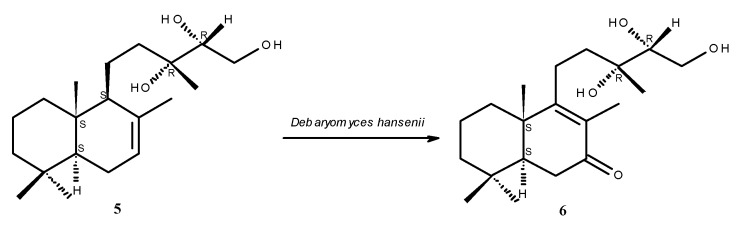
Microbiological transformation of labdane **5** into **6** [33].

**Figure 4:**
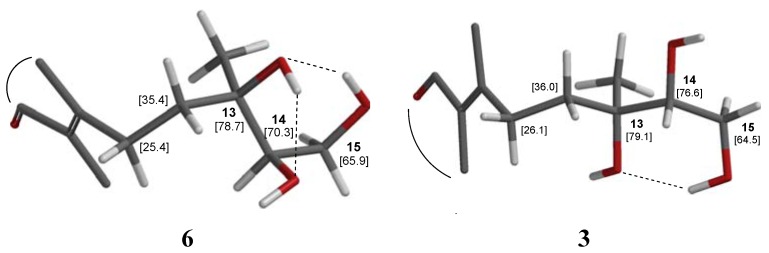
Conformations and hydrogen bridges of labdane **6** (left partial structure) and *ent*-labdane **3** (right fragment) according to DFT calculations with SPARTAN^®^. The carbon shifts were calculated for solutions in CH_2_Cl_2_.

A labdane keto-triol **6** quite similar to **3** was obtained [33] after a microbiological transformation of 13*R*,14*R*,15-trihydroxylabd-7-ene **5** (Figure 3), a diterpene commonly found in *Madia* species [34,35], as well as in *Blepharizona plumosa* [36]; **5** is a diastereomer of the *ent*-labdan **2** isolated from *G. glutinosum*. As expected, the NMR values of the pseudoenantiomeric labdane keto-triol **6** [33] are very similar to those of **3**. The main difference between both compounds is the chemical shift of C-14 (δ_C_ = 77.2 in **3**, δ_C_ = 74.9 in the reported diastereomeric labdane **6**). This seems to be due to a change in the chirality of C-13, which in **2** and **3** is (*S*) but (*R*) in the labdane diterpene from *Madia* species. The configurations on the carbons 13 (*R*) and 14 (*R*) in labdane **6** (Figure 4) would allow hydrogen bonding between the hydroxyl groups that probably diminishes the electronegative effect of the involved oxygen atoms and might result in increased shielding on these carbons and consequently leads to the high field chemical shift of C-14 (δ_C_ = 74.9). In *ent*-labdane **3** hydrogen bonding (like in labdane **6**) should be expected preferentially between 13-OH and 15-OH which thus leads to the low field chemical shift of C-14 (δ_C_ = 77.2). This assumption is nicely confirmed by DFT calculations [37] with predicted shifts close to the experimental values (Figure 4).

There are some differences in the chemical shifts assignments reported by Haridy *et al.* [33] for the ^13^C-NMR spectrum of labdane **6** for the positions 1–3 (δ_C_ = 18.6, 41.3 and 35.9) in comparison with the signals of our *ent*-labdane **3** (δ_C_ = 35.9, 18.6, 41.2). The hydrogen and carbon connectivities in **3** were deduced from the ^1^H-^1^H COSY, HSQC and HMBC spectra (Figure 2). The attachment of methyl groups 18 and 19 at C-4 was clearly illustrated by the cross-peak of methyl signals in the HMBC spectrum at δ_C_ = 32.5 and 21.3 with H-5 represented by a multiplet centered at δ_H_ = 1.67 and with CH_2_-3 represented by two multiplets at δ_H_ = 1.16 and 1.40 (HSQC confirms δ_C_ = 41.2 for C-3). Likewise, the attachment of methyl 20 at C-10 was determined by the cross-peak of the signal cat δ_C_ = 18.1 with H-5 and with CH_2_-1 represented by two multiplets at δ_H_ = 1.27 and 1.85 (δ_C_ = 35.9 by HSQC for C-1). Our assignments for the chemical shifts of the methylene groups in the positions 1–3 of compound **3** are in agreement with other literature values reported for the labdane skeleton [24,31,32] and are also reproduced by prediction programs like the ACD/CNMR Predictor 89 [38].

Finally, from the methanol extract of *G. glutinosum*, D-perseitol (**4**), a known naturally occurring heptitol [39,40], was isolated for the first time from this plant. The physical and optical properties of the isolated perseitol are sufficiently different from the diasteromeric heptitols D-volemitol and D-β-sedoheptitol, isolated from other plants [41,42].

### 2.2. Cytotoxicity Activity

**Figure 5:**
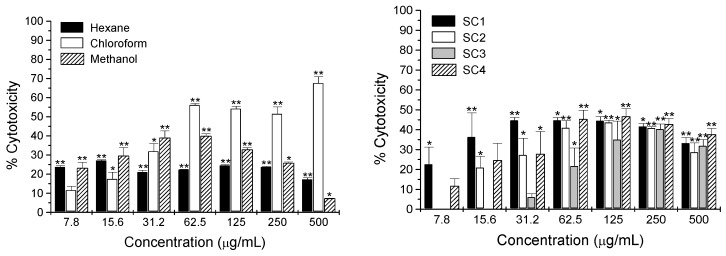
Cytotoxicity of extracts and fractions SC1 to SC4 from hexane extract of *Gymnosperma glutinosum* against L5178Y-R cells *in vitro*, as compared with untreated control (culture medium). Data represent means ± SD of triplicate determinations from 3 independent experiments. Optical density at 540 nm for untreated cells was 0.53 ± 0.06.

The cytotoxic activity of the extracts of *G. glutinosum* and compounds **1**–**4** was determined by using the *in vitro* L5178Y-R lymphoma murine model [27]. Viability of L5178Y-R tumor cells was significantly (*p* < 0.05) reduced by *G. glutinosum* extracts (Figure 5); hexane and methanol extracts caused significant cytotoxicity of 24% and 23%, respectively, at the lowest concentration tested (7.8 µg/mL). In addition, fractions SC1 and SC4 obtained from the hexane extract after column chromatography over silica gel, showed the highest cytotoxicity of 45% and 27%, respectively, at 31.2 µg/mL (Figure 5).

**Figure 6:**
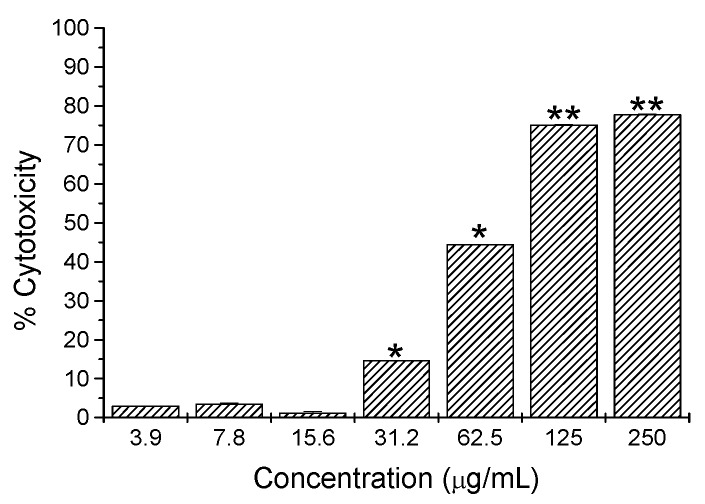
Cytotoxicity of pure compound **2** from *Gymnosperma glutinosum* against L5178Y-R cells *in vitro*, as compared with untreated control (culture medium). Data represent means ± SD of triplicate determinations from three independent experiments. Optical density at 540 nm for untreated cells was 0.53 ± 0.06.

The bioassay guided purification of fractions SC1 and SC4 from the hexane extract resulted in the isolation and identification of three compounds that were tested for cytotoxic activity against L5178Y-R cells, from which only compound **2** was observed to be cytotoxic. Hentriacontane (**1)** and the new *ent*-labdane **3** showed not significant cytotoxicity (up to 33% cytotoxicity; data not shown), whereas the *ent*-labdane **2** showed significant (*p* < 0.05) and concentration-dependent cytotoxicity (up to 78%) against L5178Y-R cells at concentrations ranging from 7.8 to 250 µg/mL (Figure 6). The heptitol **4** isolated from the methanolic extract did not show cytotoxicity at any concentration tested.

## 3. Experimental

### 3.1. General

Melting points were determined on an Electrothermal 9100 apparatus (Electrothermal Engineering Ltd., Southend on Sea, Essex, UK). Optical rotations were recorded on a PolyScience Polarimeter Model SR-6 and on a Perkin Elmer Polarimeter Model 241. IR spectra were recorded on a Varian 1000 FT-IR spectrometer using an ATR accessory. NMR spectra were measured on a Bruker DPX Spectrometer (^1^H, 400 MHz; ^13^C, 100 MHz). ESI HR mass spectra were measured on a Bruker FTICR 4.7 T Mass spectrometer. EI MS was recorded on a Finnigan MAT 95 spectrometer (70 eV). TLC was carried out on pre-coated silica gel glass plates 5 × 10 cm (Merck silica gel 60 F_254_, Darmstadt, Germany). Normal phase column chromatography was performed on silica gel (60–200 mesh) purchased from J. T. Baker (Phillipsburg, NJ, USA). Size-exclusion chromatography was performed on Sephadex LH-20 (Lipophilic Sephadex, Amersham Biosciences Ltd; purchased from Sigma-Aldrich Chemie, Steinheim, Germany).

### 3.2. Plant Material

Aerial parts of *G. glutinosum* were collected in Escobedo, Nuevo León, México, in July 2003 and identified by Maria del Consuelo González. A voucher specimen (No. 024247) was deposited at the Herbario de la Facultad de Ciencias Biológicas (UANL), Nuevo León, México.

### 3.3. Extraction and Isolation

Ground and dried leaves of *Gymnosperma glutinosum* (200 g) were sequentially extracted by maceration with 1 L *n*-hexane, chloroform and methanol. After filtration, the solvent was removed under reduced pressure to yield 20.5, 39.9 and 39.4 g of extract, respectively. Each extract was analyzed for cytotoxic activity (Figure 5). The hexane extract, which was more active than the others at low concentrations, was divided into two portions of ca. 10 g and each of them chromatographed on a silica gel (190 g) column (160 × 3 cm) and eluted with stepwise gradients of *n*-hexane-chloroform (100:0, 90:10, 80:20, 70:30, 60:40, 50:50, 40:60, 30:70, 20:80, 10:90, 0:100 v/v, each 400 mL), chloroform-ethyl acetate (90:10, 80:20, 70:30, 60:40, 50:50, 40:60, 30:70, 20:80, 10:90, 0:100 v/v, each 400 mL), and finally with 800 mL methanol. A total of 184 subfractions (50 mL) were collected for each column and combined on the basis of their TLC (*n*-hexane-CHCl_3_ 1:1) profiles into four main fractions as follows: subfractions 1–43 SC1 (1.6 g), subfractions 44–78 SC2 (2.6 g), subfractions 79–104 SC3 (2.4 g) and subfractions 105–184 SC4 (5.9 g). These main fractions, containing the non-polar to the more polar compounds, were used for cytotoxicity assays (Figure 5). Fractions SC1 and SC4 showed the best activity against L5178Y–R and were submitted to additional fractionation. Fraction SC1 was chromatographed again on a silica gel (20 g) column (36 × 2 cm) using a stepwise gradient solvent system consisting of *n*-hexane-chloroform (100:0, 90:10, 80:20, 70:30, 60:40, 50:50, 40:60, 30:70, 20:80, 10:90, 0:100 v/v, each 50 mL), chloroform-ethyl acetate (90:10, 80:20, 70:30, 60:40, 50:50, 40:60, 30:70, 20:80, 10:90, 0:100 v/v, each 50 mL), and finally with 100 mL methanol. A total of 115 subfractions (10 mL) were collected, combined on the basis of their TLC (*n*-hexane-CHCl_3_ 1:1) profiles into eight main fractions as follows: A (subfractions 1–6), B (subfractions 7–21), C (subfractions 22–34), D (subfractions 35–40), E (subfractions 41–49), F (subfractions 50–61), G (subfractions 62–70) and H (subfractions 71–115). Fraction A yielded 200 mg hentriacontane (**1**) whereas fractions B to H were still complex mixtures (NMR) of *n***-**alkanes and probably diterpenes. Fraction SC4 was also chromatographed on a silica gel (190 g) column (160 × 3 cm) using a stepwise gradient solvent system consisting of chloroform-ethyl acetate (100:0, 90:10, 80:20, 70:30, 60:40, 50:50, 40:60, 30:70, 20:80, 10:90, 0:100 v/v, each 400 mL), and finally with 800 mL methanol. A total of 260 subfractions (20 mL) were collected, combined on the basis of their TLC (CHCl_3_–EtOAc, 1:1) profiles into six main fractions as follows: I (subfractions 1–36), J (subfractions 37–60), K (subfractions 61–92), L (subfractions 93–143), M (subfractions 143–182) and N (subfractions 183–260). Fraction L produced almost pure (+)-13*S*,14*R*,15-trihydroxy-*ent*-labd-7-ene (**2**) whereas fraction M rendered (−)-13*S*,14*R*,15-trihydroxy-7-oxo-*ent*-labd-8(9)-ene (**3**). Both diterpenes were subjected to additional purification on a Sephadex LH-20 (50 g) column (160 × 1.5 cm), using MeOH as eluent. For diterpene **2** a total of 240 subfractions (10 mL) were collected and after combining subfractions 17–20, previously purity judged from TLC (CHCl_3_–EtOAc, 1:1), 2.5 g of (+)-13*S*,14*R*,15-trihydroxy-*ent*-labd-7-ene (**2**) were obtained. For diterpene **3** only 190 subfractions (10 mL) were collected. From subfractions 14–18, combined on the basis of their TLC (CHCl_3_–EtOAc, 1:1) profiles, 600 mg of (−)-13*S*,14*R*,15-trihydroxy-7-oxo-*ent*-labd-8(9)-ene (**3**) were obtained.

After cooling, an amorphous precipitate was formed from the methanol extract, which was collected and stirred in 30 mL cold methanol for 24 h. After filtration, 250 mg of D-glycero-D-galactoheptitol (**4**) were recovered as a white powder.

*(−)-13*S*,14*R*,15-Trihydroxy-7-oxo-*ent*-labd-8(9)-ene* (**3**). Colorless oil; *R_f_* = 0.18 (CHCl_3_–EtOAc, 1:1); [α]_D_^20^: −16.5 (c 2.0, CHCl_3_); IR (ATR): ν_max_ = 3370, 2915, 1710, 1690, 1640, 1462, 1372, 1018, 720 cm^−1^; ^1^H- and ^13^C-NMR data in CDCl_3_, see Table 1; ESI-MS (+)-mode: *m/z* = 361 [M+Na]^+^, (−)-mode: *m/z* = 337 [M−H]^−^; (+)-ESI HR MS: *m/z* = 339.25300 [M+H]^+^ (calcd. for C_20_H_35_O_4_: 339.25298).

### 3.4. Cytotoxicity Assay

#### 3.4.1. Reagents and Culture Medium

Penicillin-streptomycin solution, L-glutamine, and RPMI 1640 medium were obtained from Life Technologies (Grand Island, NY, USA). Fetal bovine serum (FBS), sodium dodecyl sulfate (SDS), N,N-dimethylformamide (DMF) and 3-[4,5-dimethylthiazol-2-yl]-2,5-diphenyltetrazolium bromide (MTT) were purchased from Sigma-Aldrich Co. (St. Louis, MO, USA). Vincristine sulfate salt (97.5%) was obtained from Vintec (Columbia S. A. de C. V., México). Extraction buffer was prepared by dissolving 20% (wt/vol) SDS at 37 °C in a solution of 50% each DMF and demineralized water, and the pH was adjusted to 4.7.

#### 3.4.2. Tumor C ell Line

The tumor cell line L5178Y-R (mouse DBA/2 lymphoma) was purchased from The American Type Culture Collection (Rockville, MD, USA) and was maintained in culture flasks with RPMI 1640 medium supplemented with 10% FBS, 1% L-glutamine, and 0.5% penicillin-streptomycin solution (referred as complete RPMI medium) at 37 °C, in a humidified atmosphere of 5% CO_2_ in air. Cellular density was kept between 10^5^ and 10^6^ cells/mL.

#### 3.4.3. Cell Preparation and Culture

The direct *in vitro* activity of extracts, active fractions and pure compounds of *Gymnosperma glutinosum* was carried out by the tetrazolium bromide (MTT) colorimetric technique [27]. Cell cultures were collected, washed three times in RPMI 1640 and adjusted to 5 × 10^4^ cells/mL with RPMI complete medium. One hundred microliters of the cell suspension were then added to flat-bottomed 96-well plates (Becton Dickinson, Cockeysville, MD, USA), containing triplicate cultures (100 µL) of *G. glutinosum* extracts, active fractions or pure compounds at various concentrations as well as positive (vincristine) and negative (culture medium) controls. The plates were then incubated for 44 h at 37 °C in 5% CO_2_ atmosphere. Next, 0.5 mg/mL of MTT were added, and the cultures were additionally incubated for 4 h. After this, 100 µL extraction buffer were added to all wells and plates were incubated for 16 h and optical densities, resulting from dissolved formazan crystals, were then read in a microplate reader (Bio-Tek Instruments, Inc., Winooski, VT, USA) at 540 nm. The results were expressed as mean ± SD of triplicate determinations from a representative experiment. All experiments were repeated at least three times with similar results. Statistical significance was assessed by one-way analysis of variance. Cytotoxicity percentage was determined as follows:


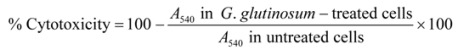


## 4. Conclusions

Hentriacontane (**1**) has already been suggested as a possible antitumor agent [43,44], however, in our case, it showed non-significant 12% cytotoxicity at 250 µg/mL (data not shown). The heptitol **4** did not show cytotoxicity at any concentration tested, although a complex with K^+^ ions has been shown to exhibit a potent inhibitory effect on [3H]-leucine incorporation for protein synthesis on Ehrlich ascites tumor cells in mice [40,45]. Our results showed significant tumor cell toxicity of the apolar *ent*-labdane **2** of up to 78% (250 µg/mL) in a concentration-dependent fashion, from concentrations as low as 7.8 µg/mL, as compared with untreated control, which may be an indication of an important antitumor activity (Figure 6), whereas *ent*-labdane **3** (polar) only caused low but significant (*p* = 0.05) 32% cytotoxicity at 250 µg/mL (data not shown). Vincristine control caused 68% to 81% cytotoxicity at concentrations ranging from 0.49 to 31.25 µg/mL respectively (data not shown). Cytotoxicity observed by the hexane extract (Figure 5) may be the result of the additive effect of all labdane-diterpenes in the extract. Separation of the polar and apolar diterpenes **3** and **2** shows that these compounds probably have to cross a lipophilic barrier in the lymphoma cell in order to induce cytotoxicity. The polar diterpene **3** is clearly significantly less effective to cross this barrier and therefore did not show significant cytotoxicity as pure compound. In our study, we decided to fractionate only the hexane extract, which was more active than the others at low concentrations (7.8 µg/mL). The chloroform extract was not successfully fractionated, however, our preliminary findings showed that this extract is a complex mixture of labdane diterpenes (5 singlets between δ 1.7 and 0.7 in the ^1^H-NMR spectrum; [24,31,32]) and highly oxygenated flavonoids (signals for aromatic protons and methoxy groups between δ 3.5 and 4.5 pin the ^1^H-NMR spectrum [18,19]; data not shown). Diterpenes of the labdane type have been reported to have a broad spectrum of biological activities [46]. Many studies have shown that labdane diterpenes exhibit significant cytotoxic and cytostatic effects against leukemic cell lines of human origin and interfere with the biochemical pathways of apoptosis and the cell cycle phases, as well as with the expression of several protooncogenes such as *c-myc* and *bcl-2* [47]. The cytotoxic activity of the *ent*-labdane diterpenes from *G. glutinosum* indicated that natural products from plants might contribute to the pool of novel anticancer drugs for which tumor resistance may not have been developed [48].

## Supplementary Materials

Supplementary materials can be accessed at: http://www.mdpi.com/1420-3049/17/9/11229/s1.
