# Supplementary material for: Bioassay-Guided Isolation and Identification of Cytotoxic Compounds from Gymnosperma glutinosum Leaves
Source: Molecules. 2012 Sep 20;17(9):11229–41. doi: 10.3390/molecules170911229 (PMC6268992; doi:10.3390/molecules170911229)
Supplement: Supplementary file 1 [file molecules-17-11229-s001.pdf]

S1: ^{1}H-NMR spectrum of compound **1** ($CDCl_3$, 400 MHz).

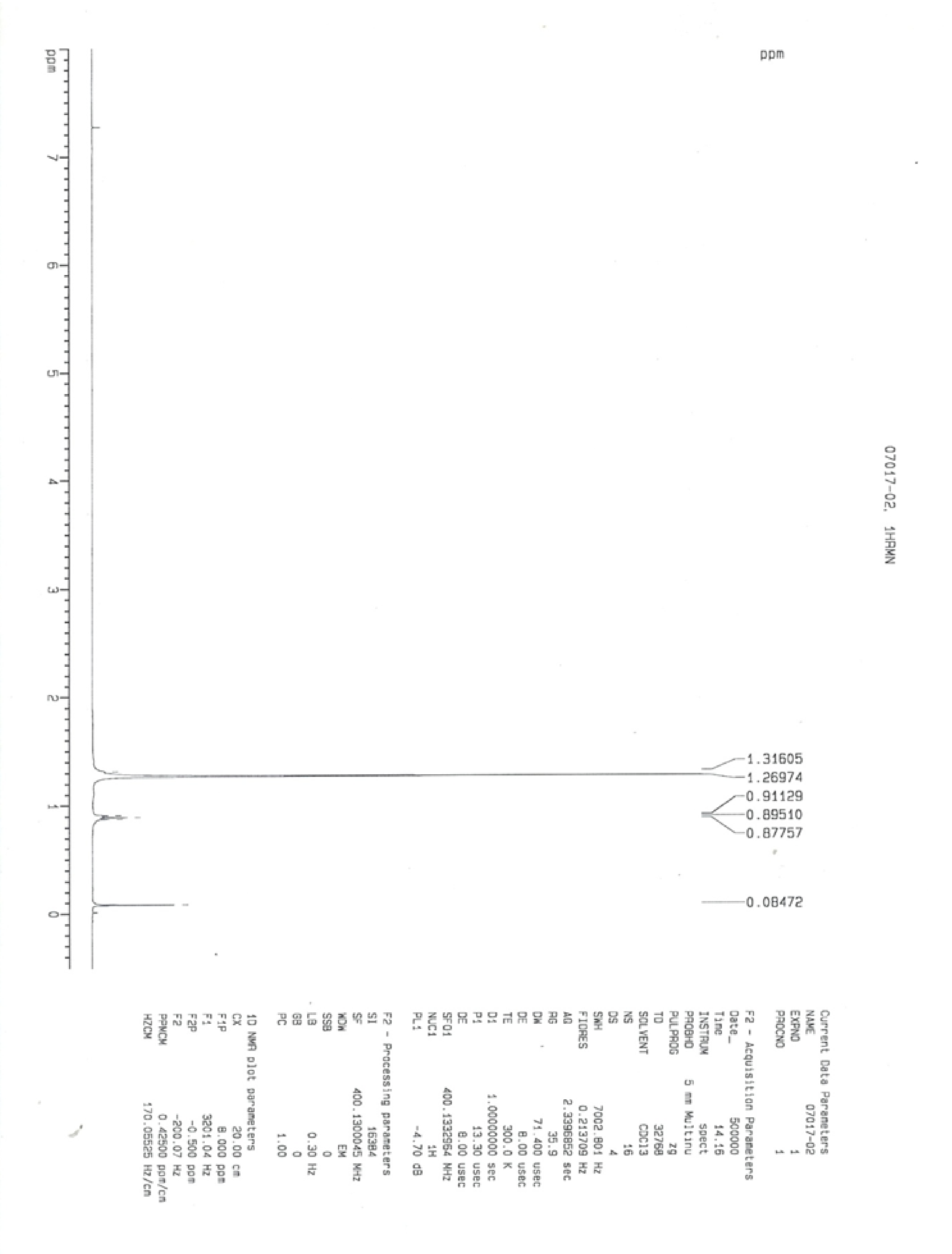

S2: ^{13}C-NMR spectrum of compound **1** ($CDCl_3$, 100 MHz).

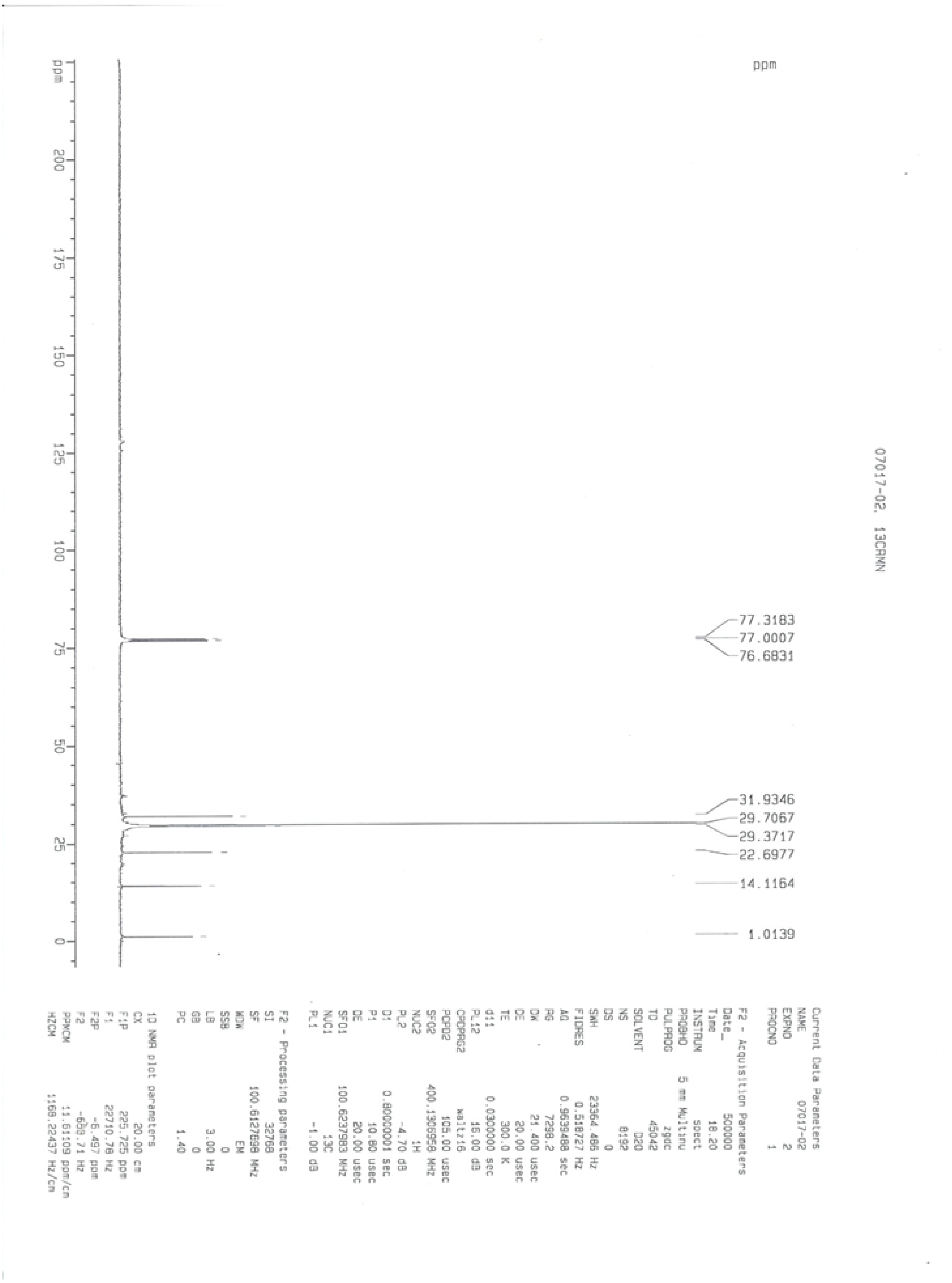

S3: DEPT spectrum of compound **1** ($CDCl_3$, 100 MHz).

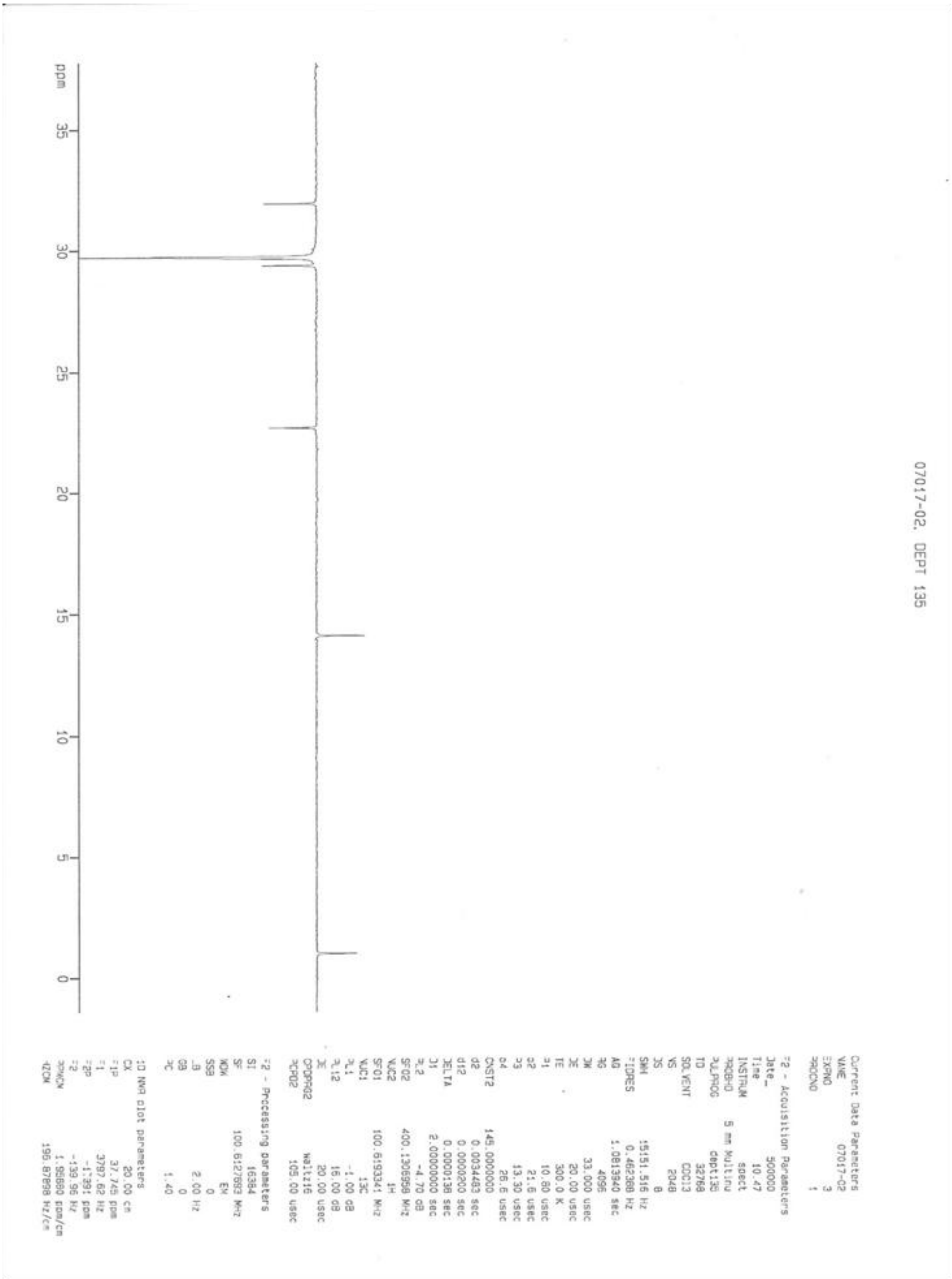

S4: ^{1}H-NMR spectrum of compound **2** ($CDCl_3$, 400 MHz).

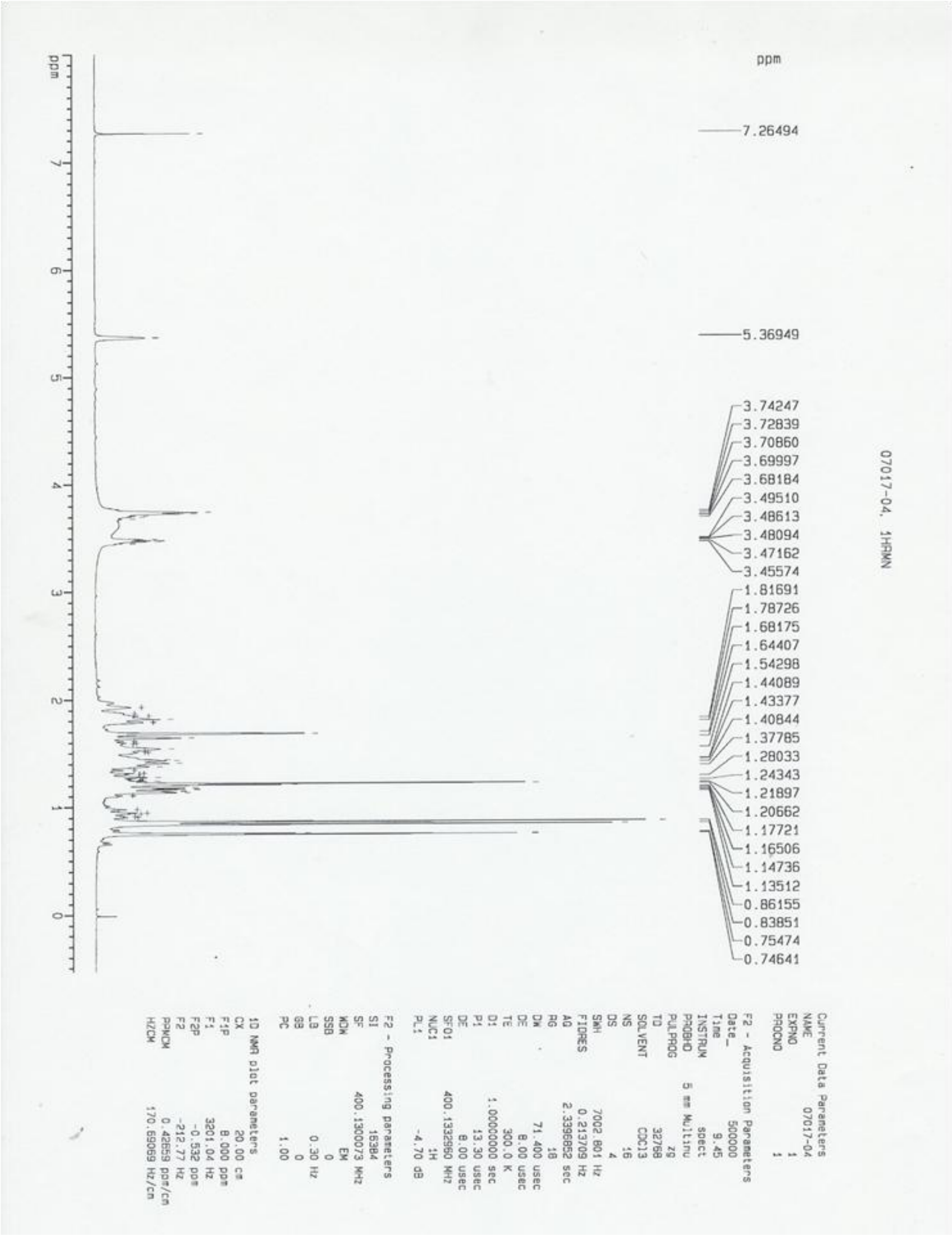

S5: ^{13}C-NMR spectrum of compound **2** ($CDCl_3$, 100 MHz).

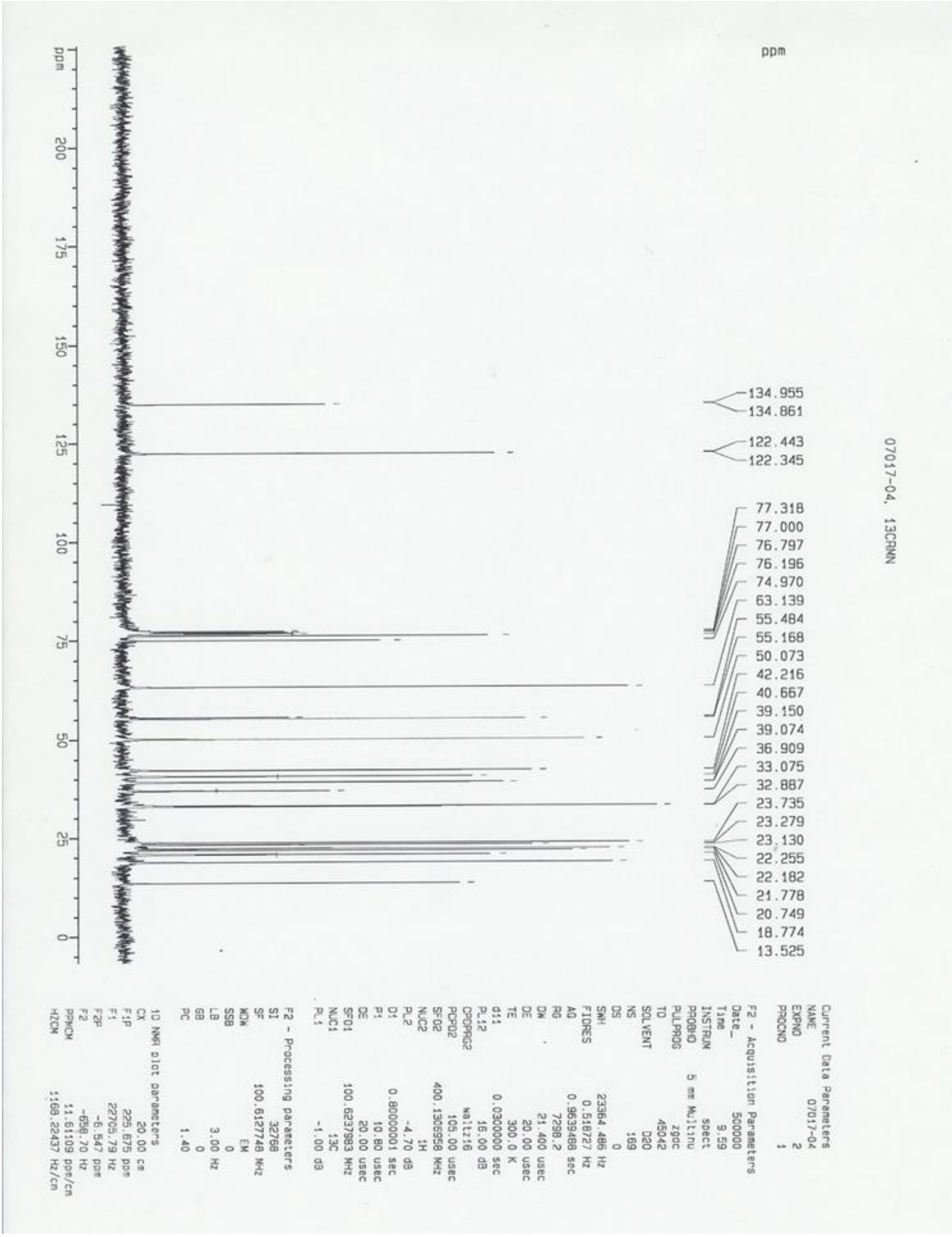

S6: DEPT spectrum of compound **2** ($CDCl_3$, 100 MHz).

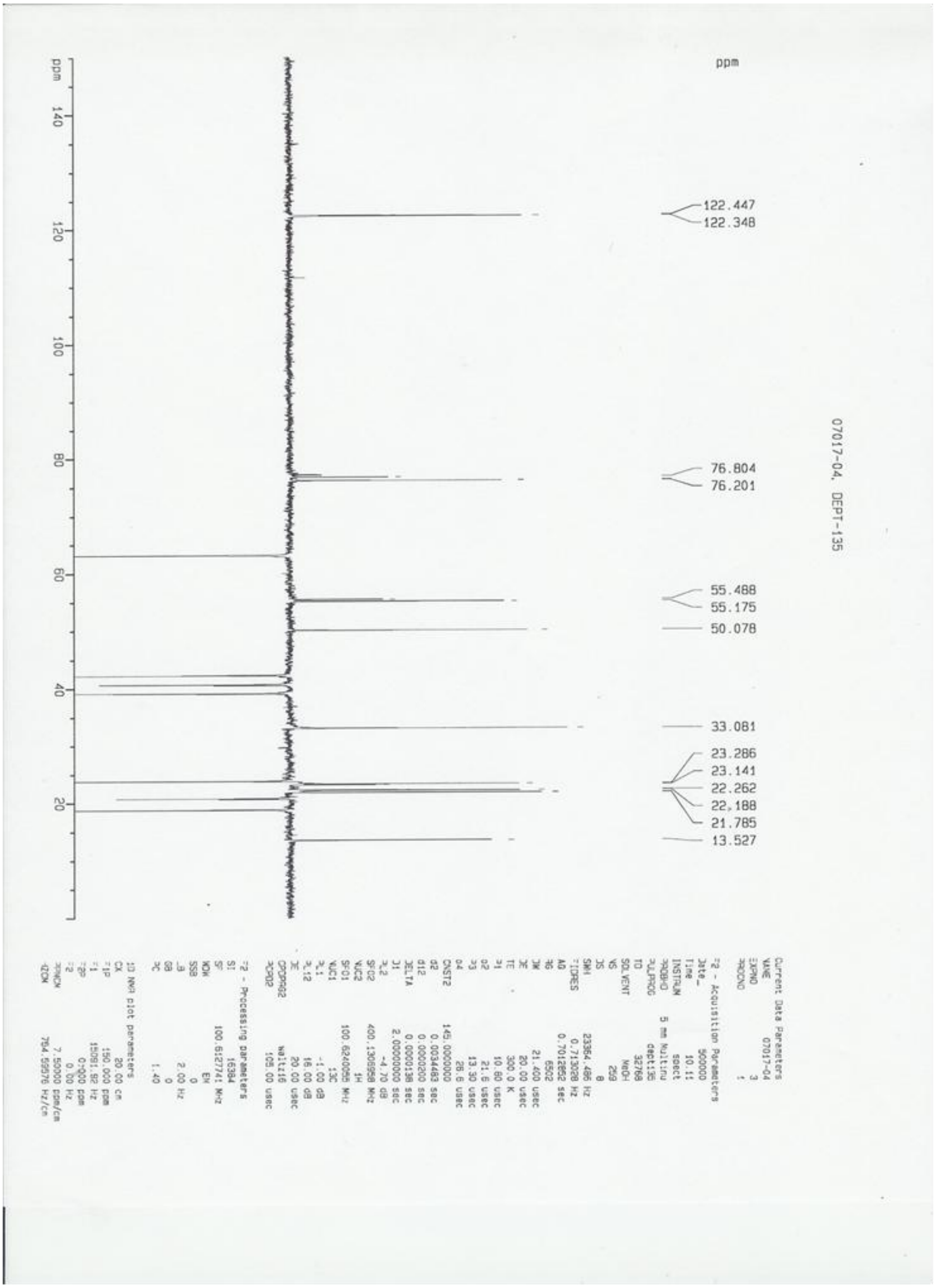

S7: COSY spectrum of compound **2** ($CDCl_3$, 400 MHz).

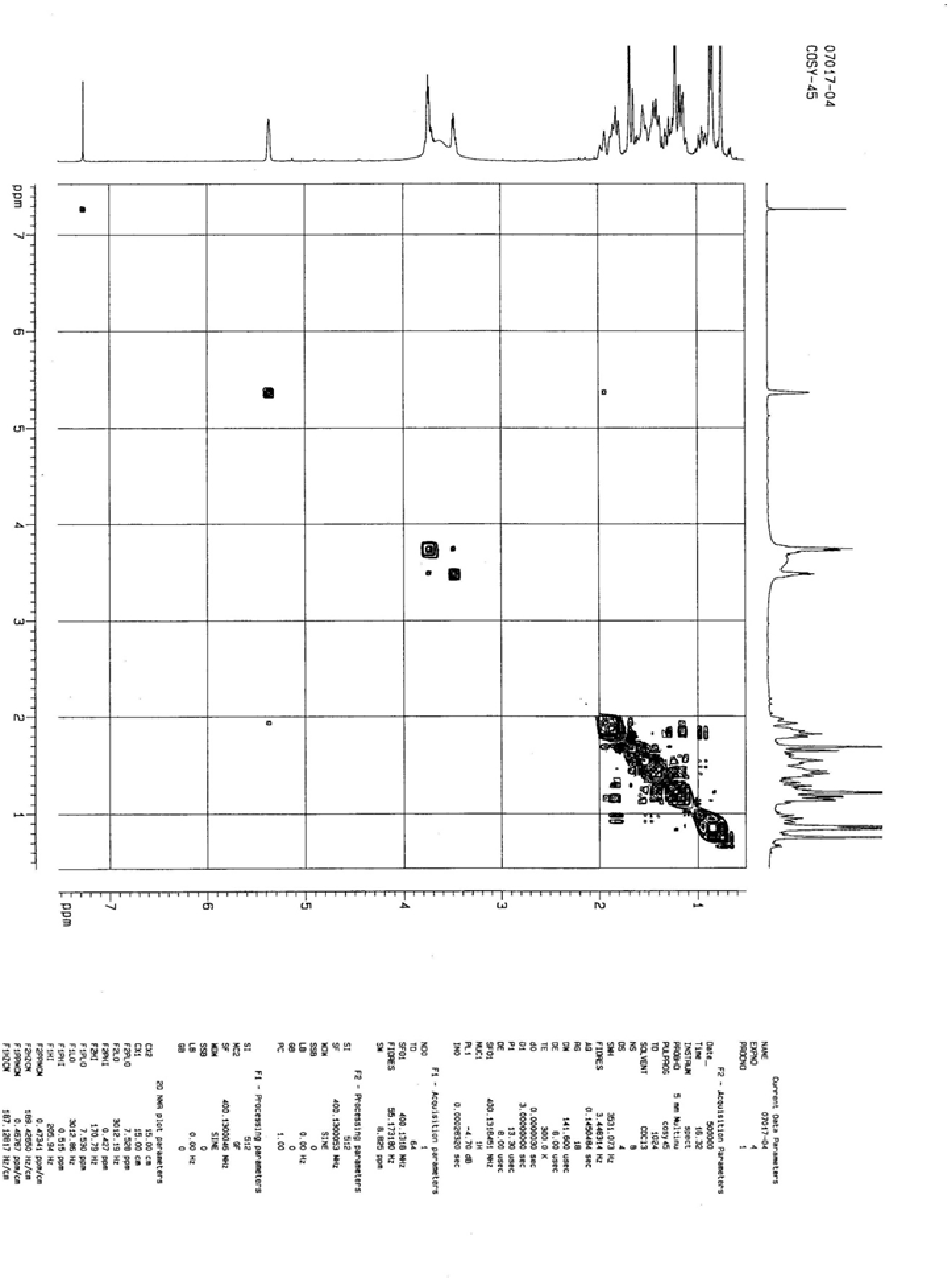

S8: HMQC spectrum of compound **2** ($CDCl_3$, 100 MHz).

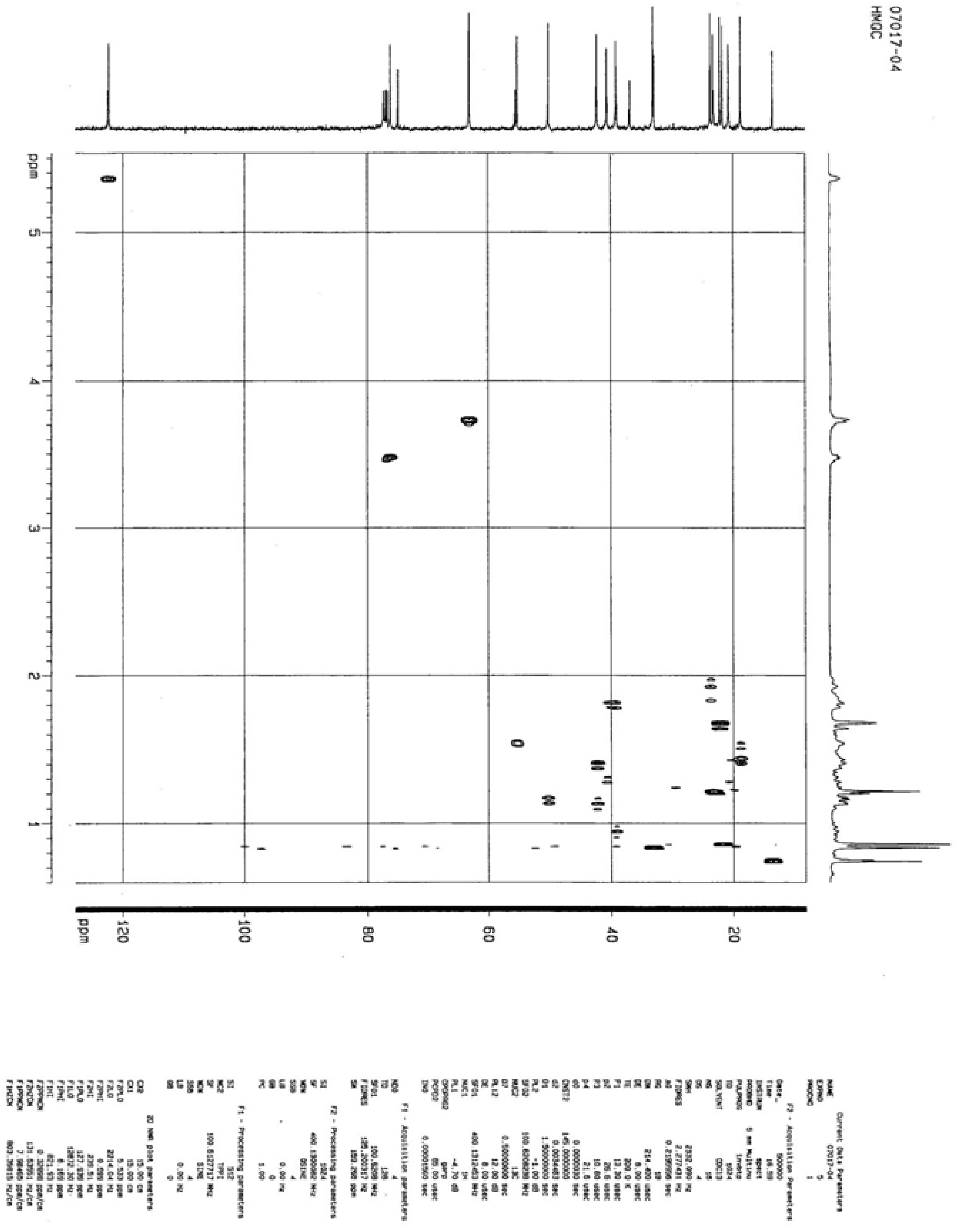

S9: ^{1}H-NMR spectrum of compound **2** (D_6-DMSO, 400 MHz).

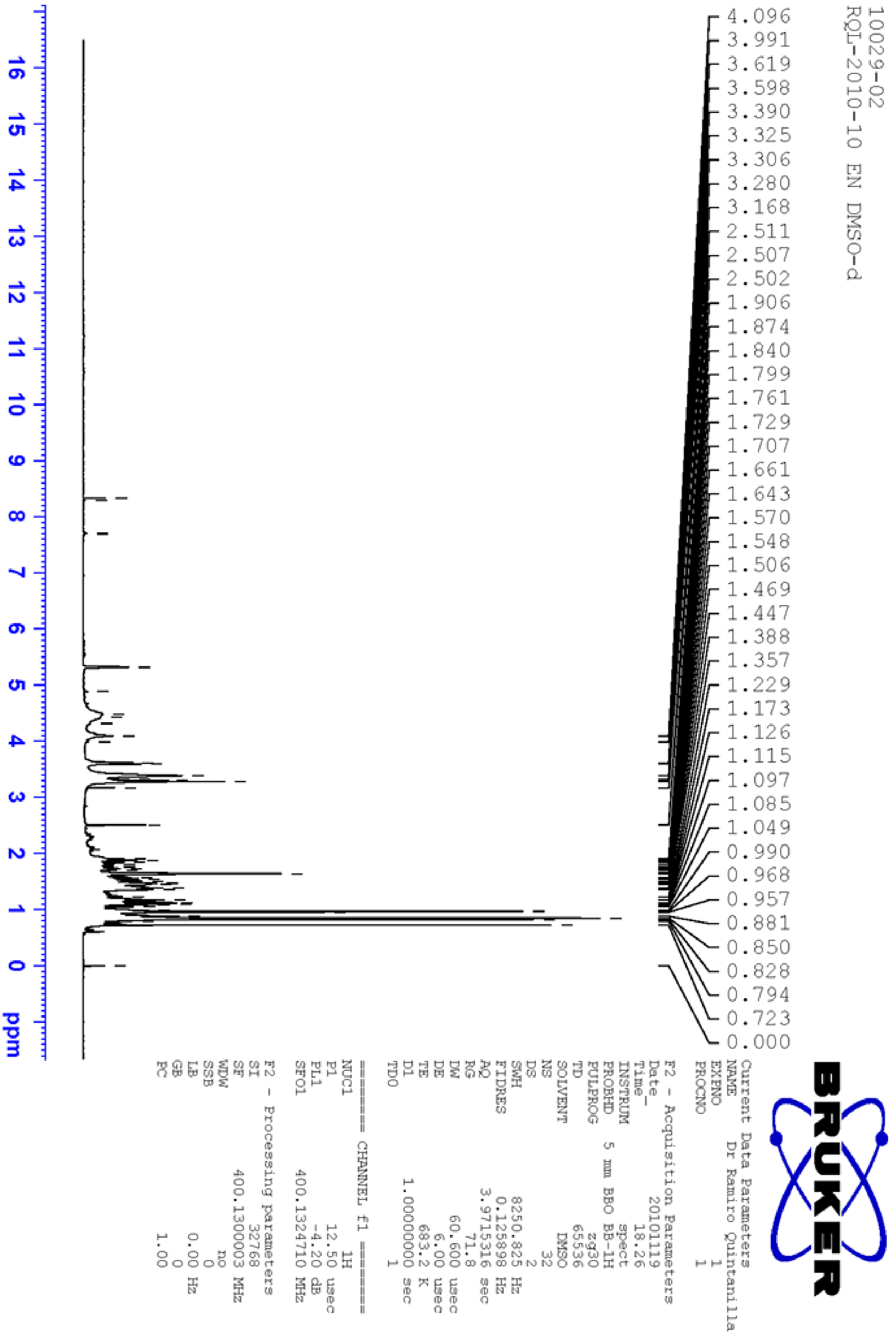

S10: ^{13}C-NMR spectrum of compound **2** (D_6-DMSO, 100 MHz).

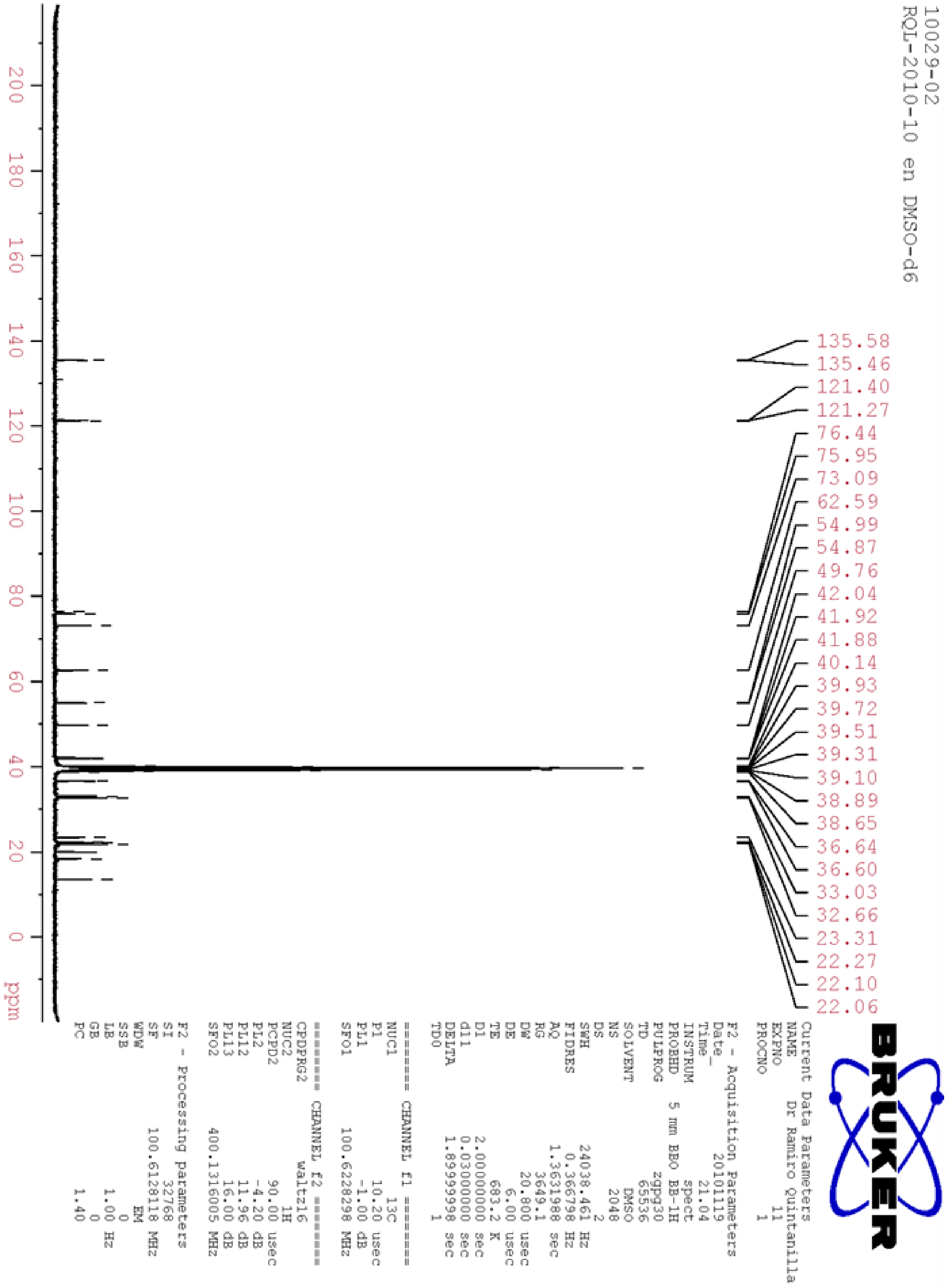

S11: DEPT spectrum of compound **2** (D_6-DMSO, 100 MHz).

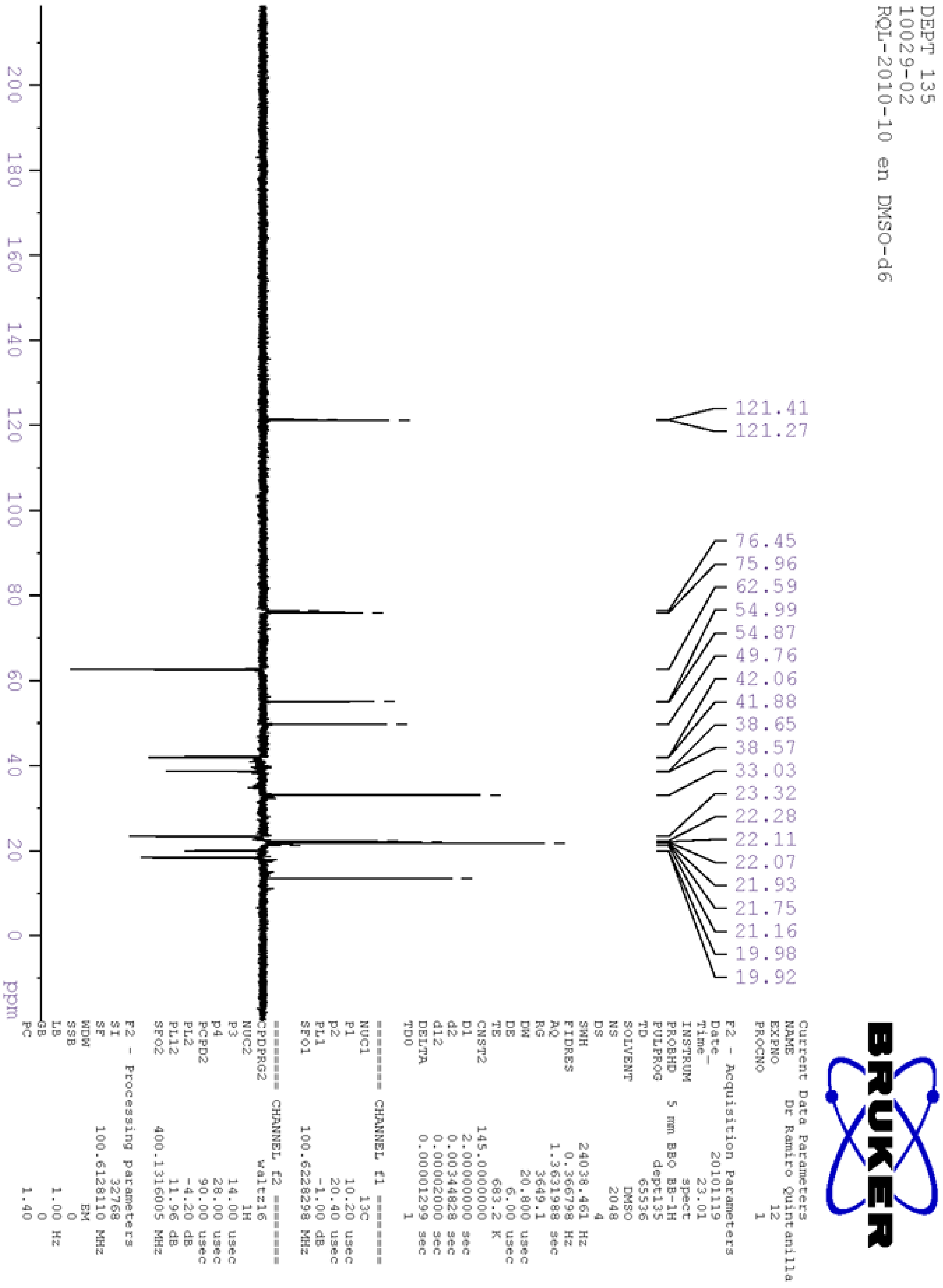

S12: COSY spectrum of compound **2** (D_6-DMSO, 400 MHz).

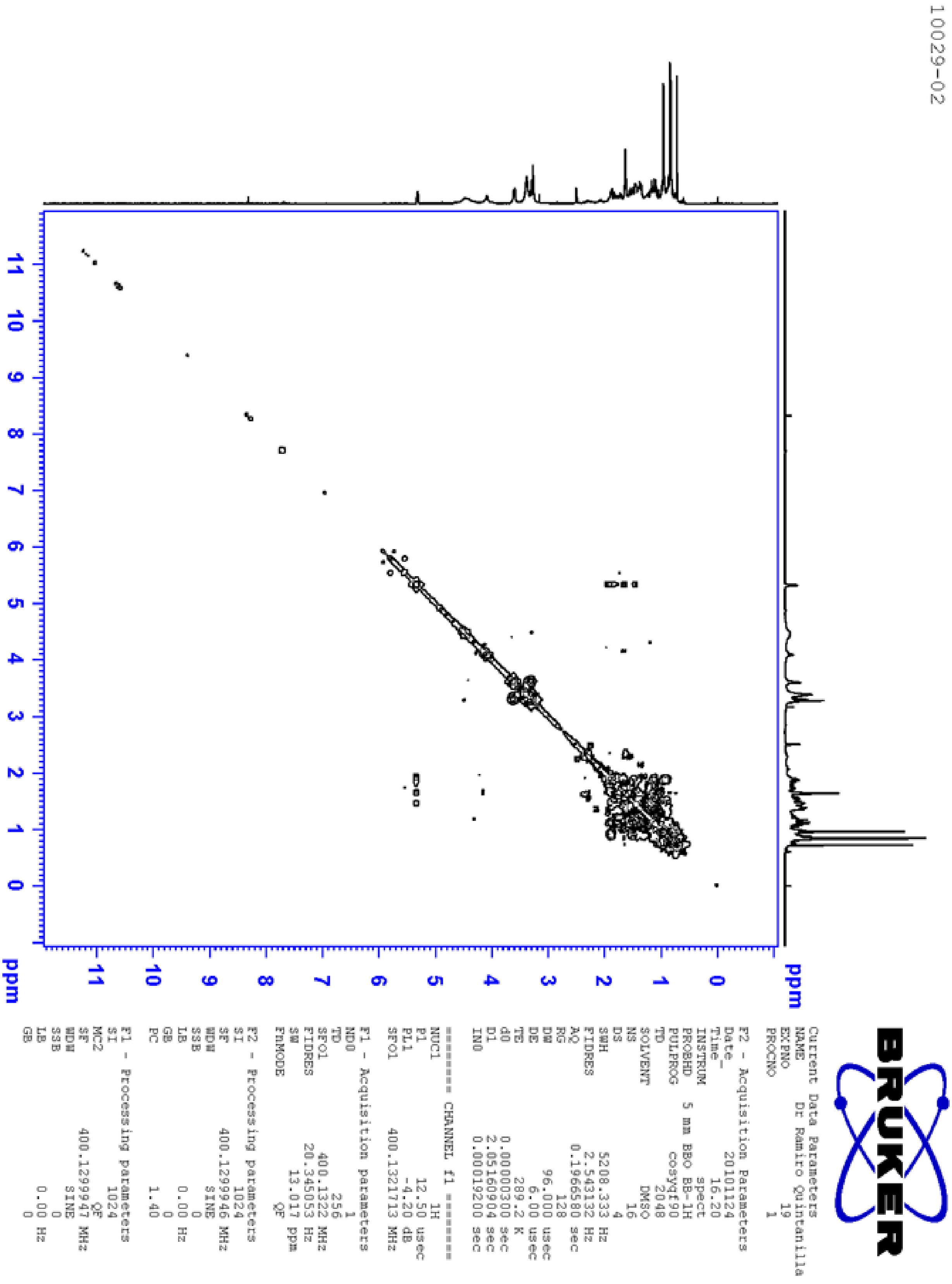

S13: HMQC spectrum of compound **2** (D_6-DMSO, 100 MHz).

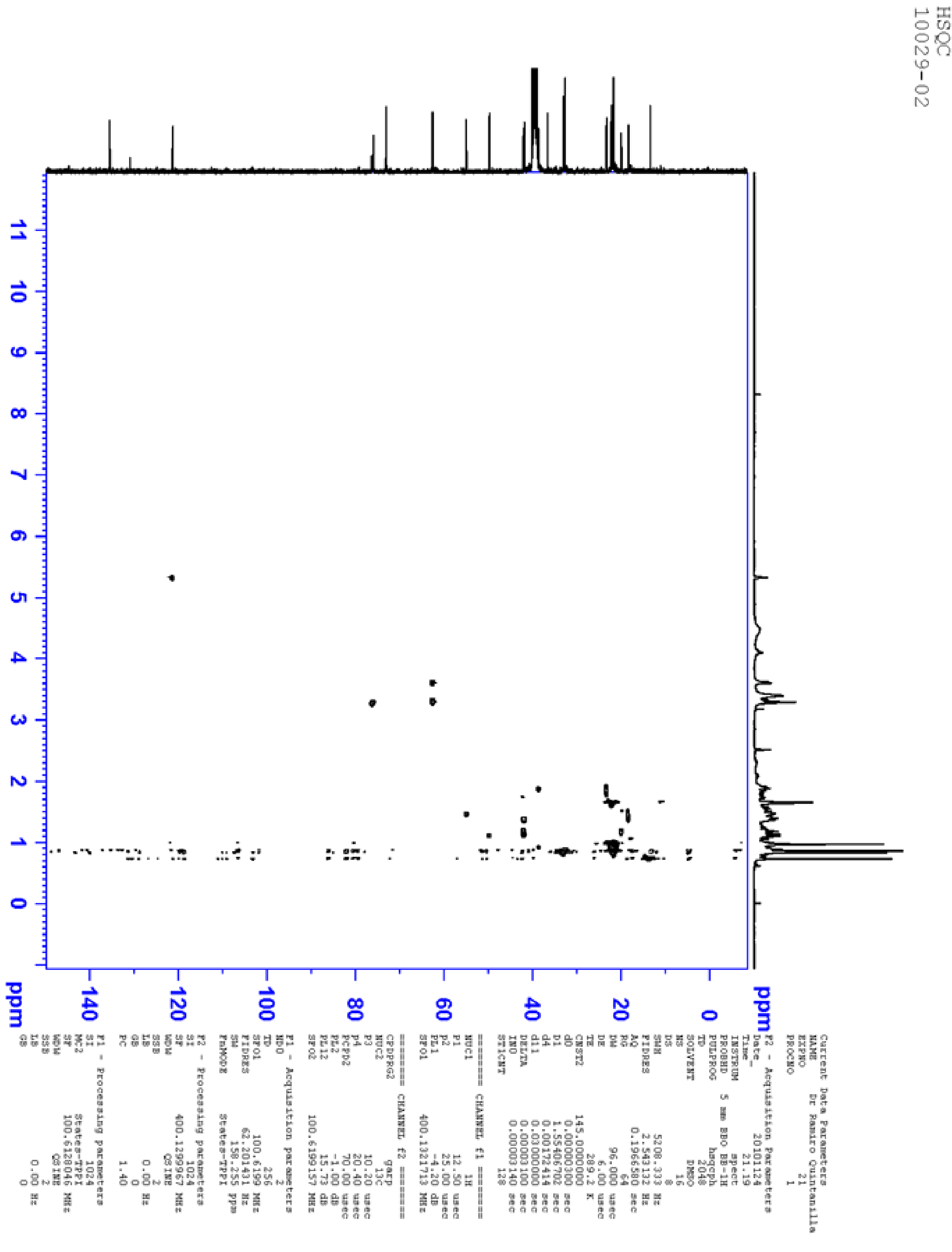

S14: ^{1}H-NMR spectrum of compound **2** after deuterium exchange (D_6-DMSO, 400 MHz).

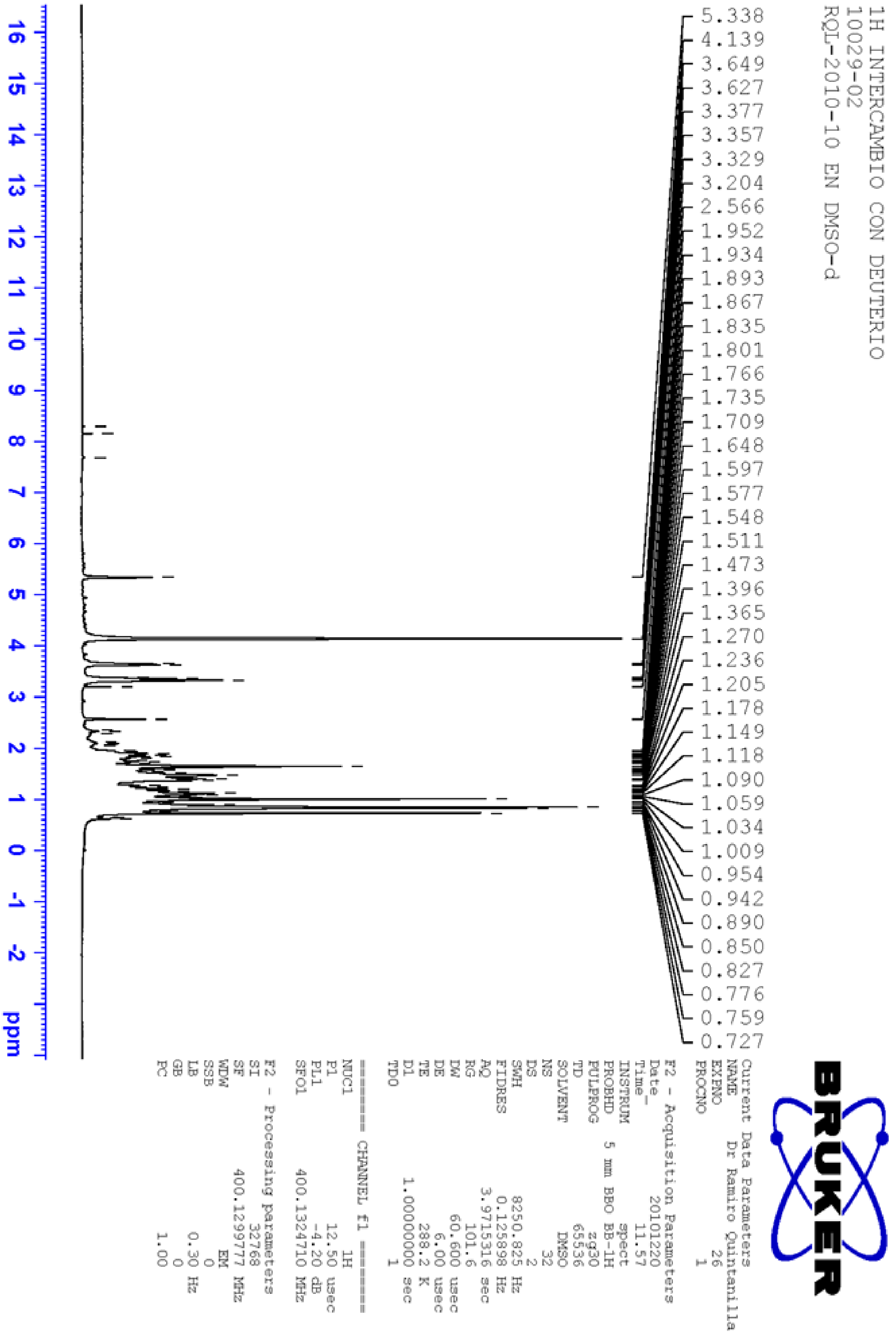

S15: ^{1}H-NMR spectrum of compound **3** ($CDCl_3$, 300 MHz).

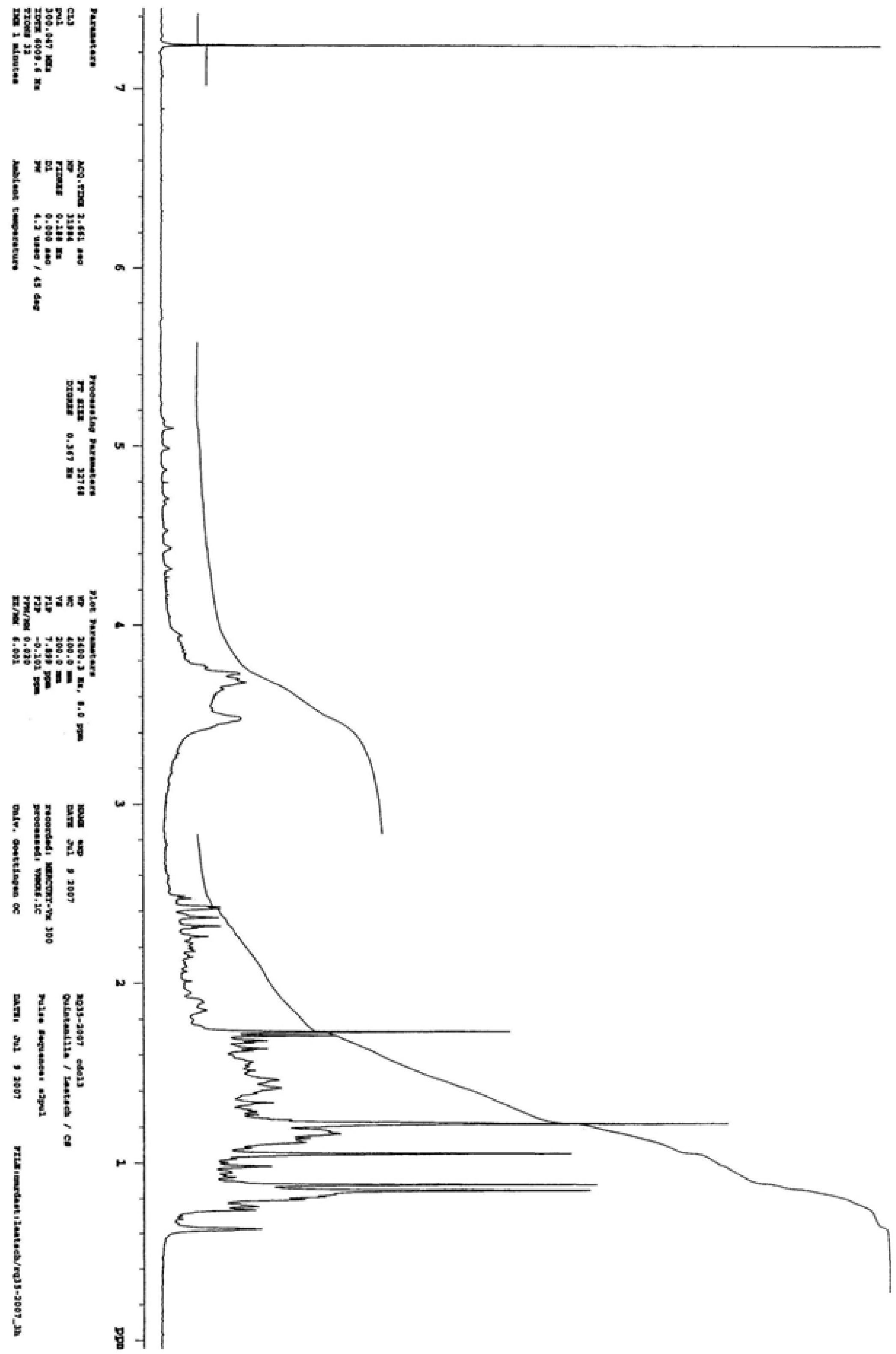

S16: ^{13}C-NMR spectrum of compound **3** ($CDCl_3$, 125 MHz).

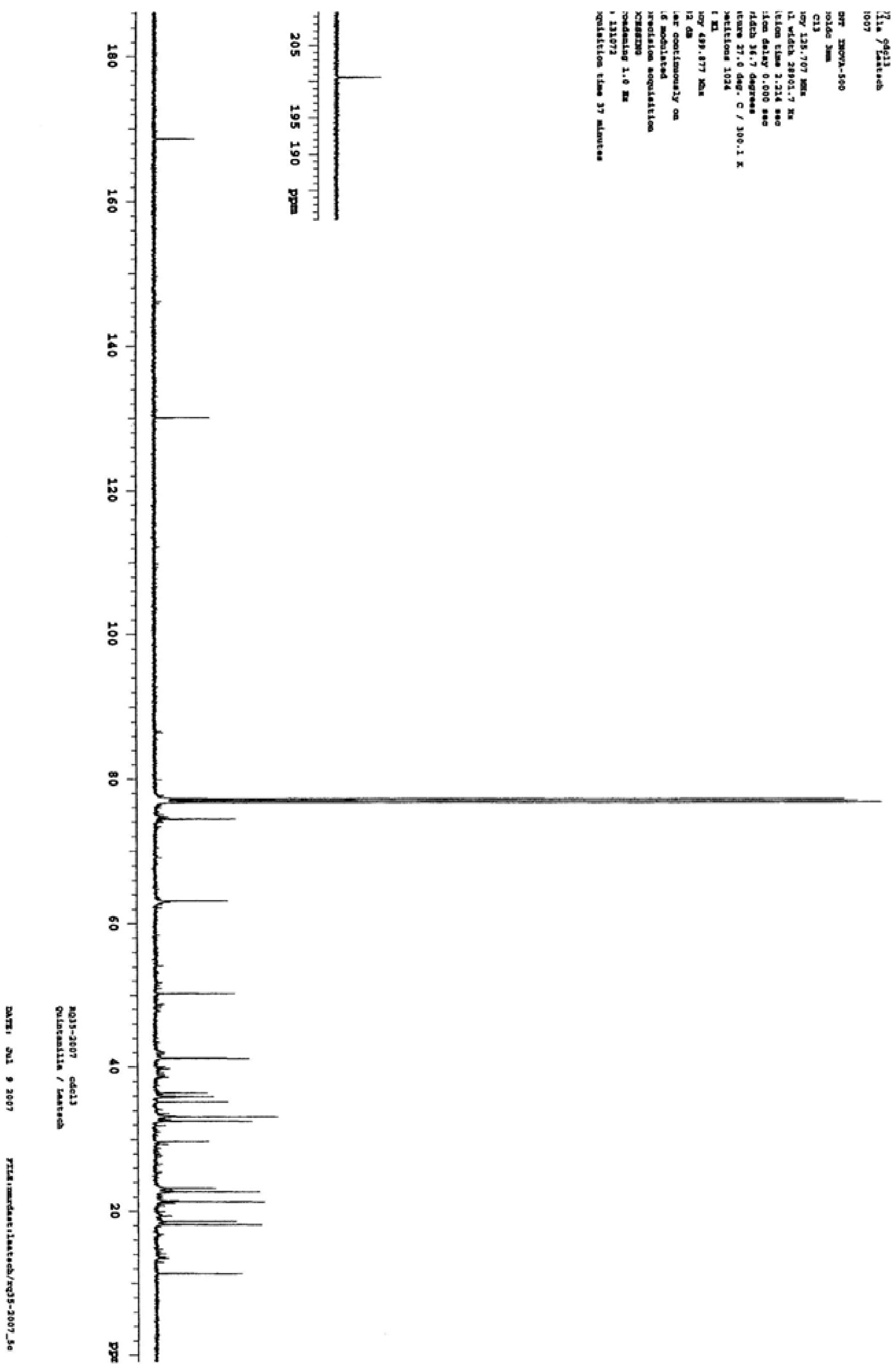

S17: APT spectrum of compound **3** ($CDCl_3$, 125 MHz).

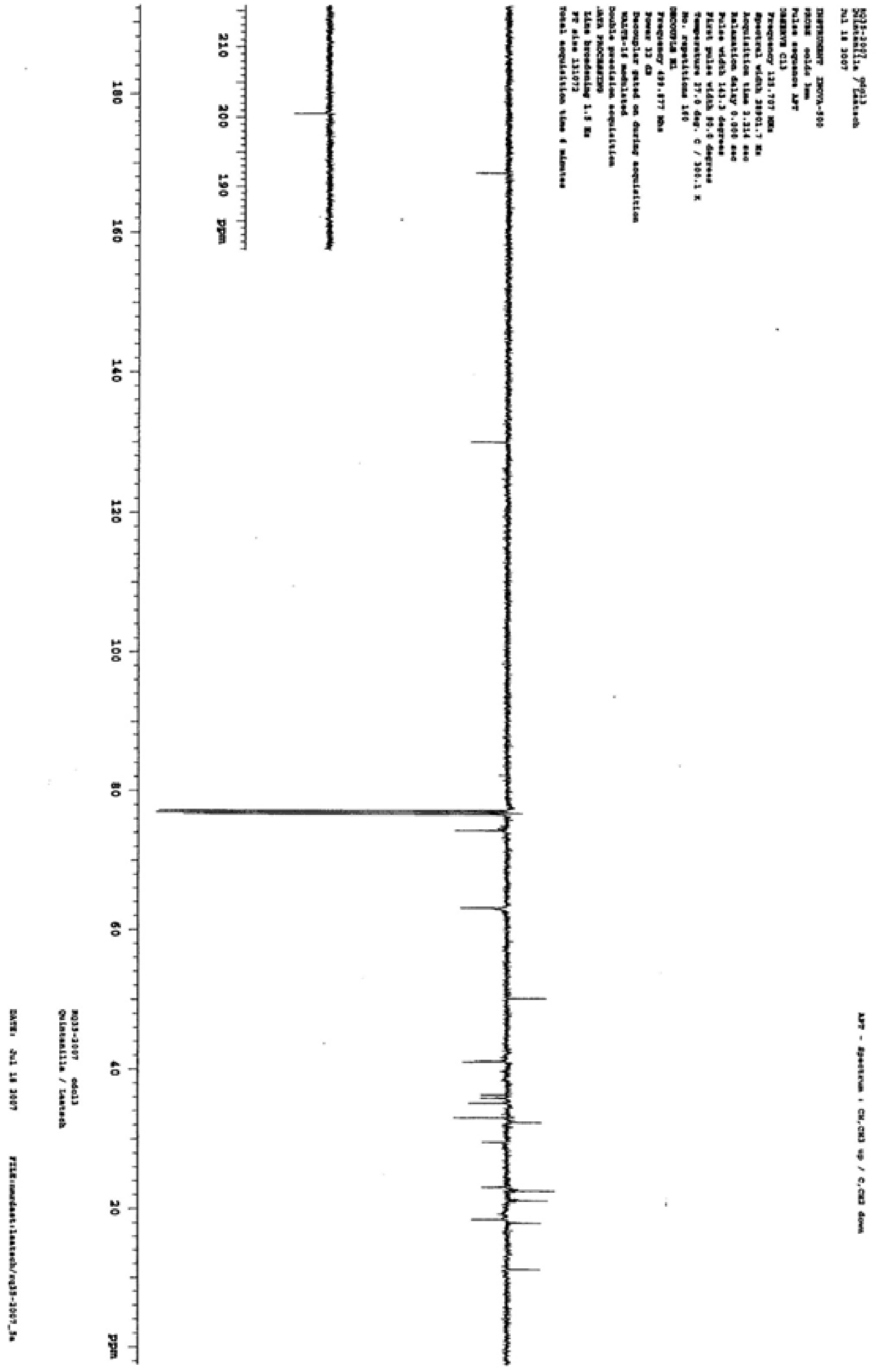

S18: COSY spectrum of compound **3** ($CDCl_3$, 300 MHz).

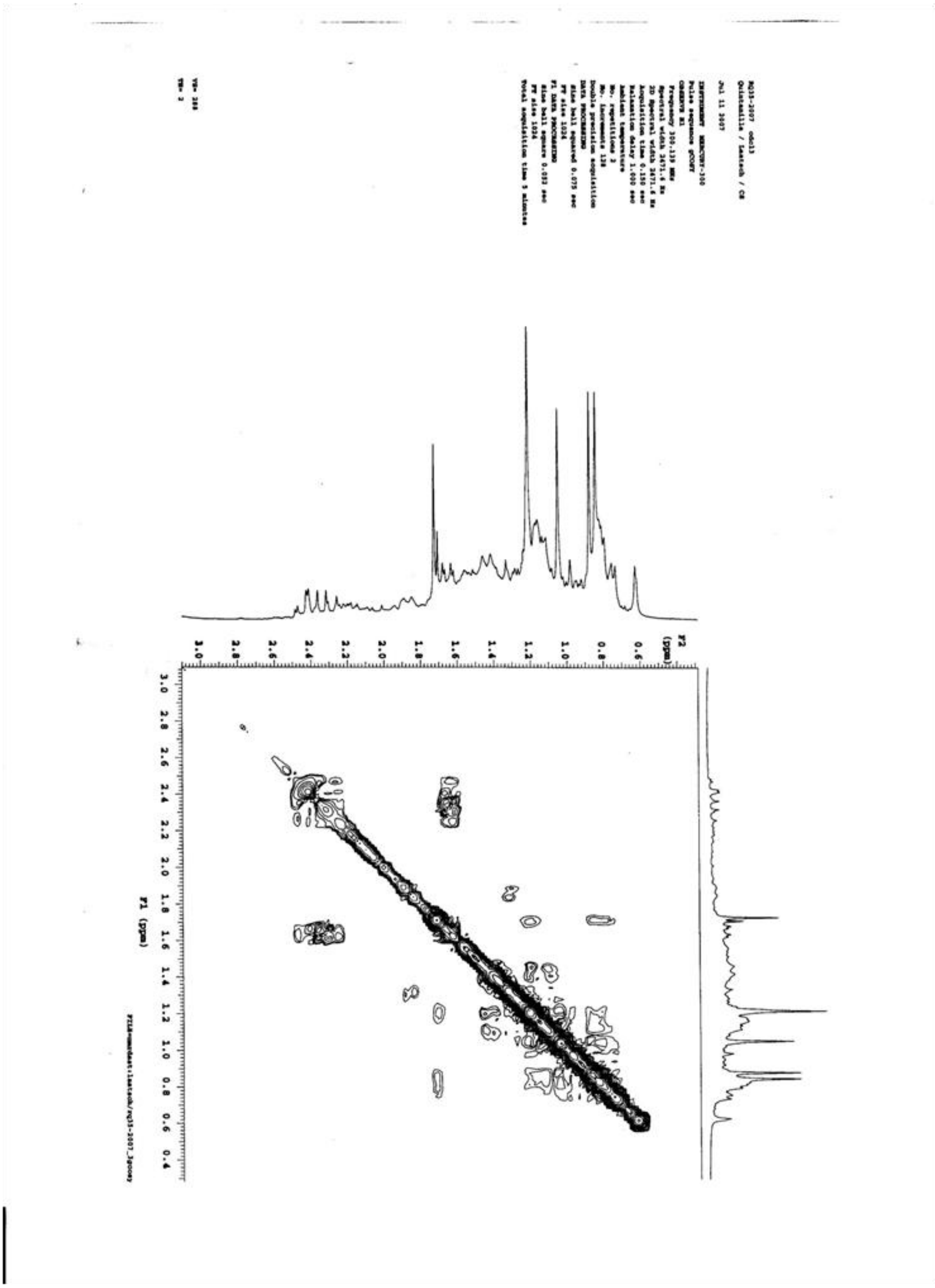

S19: HSQC spectrum of compound **3** ($CDCl_3$, 300 MHz).

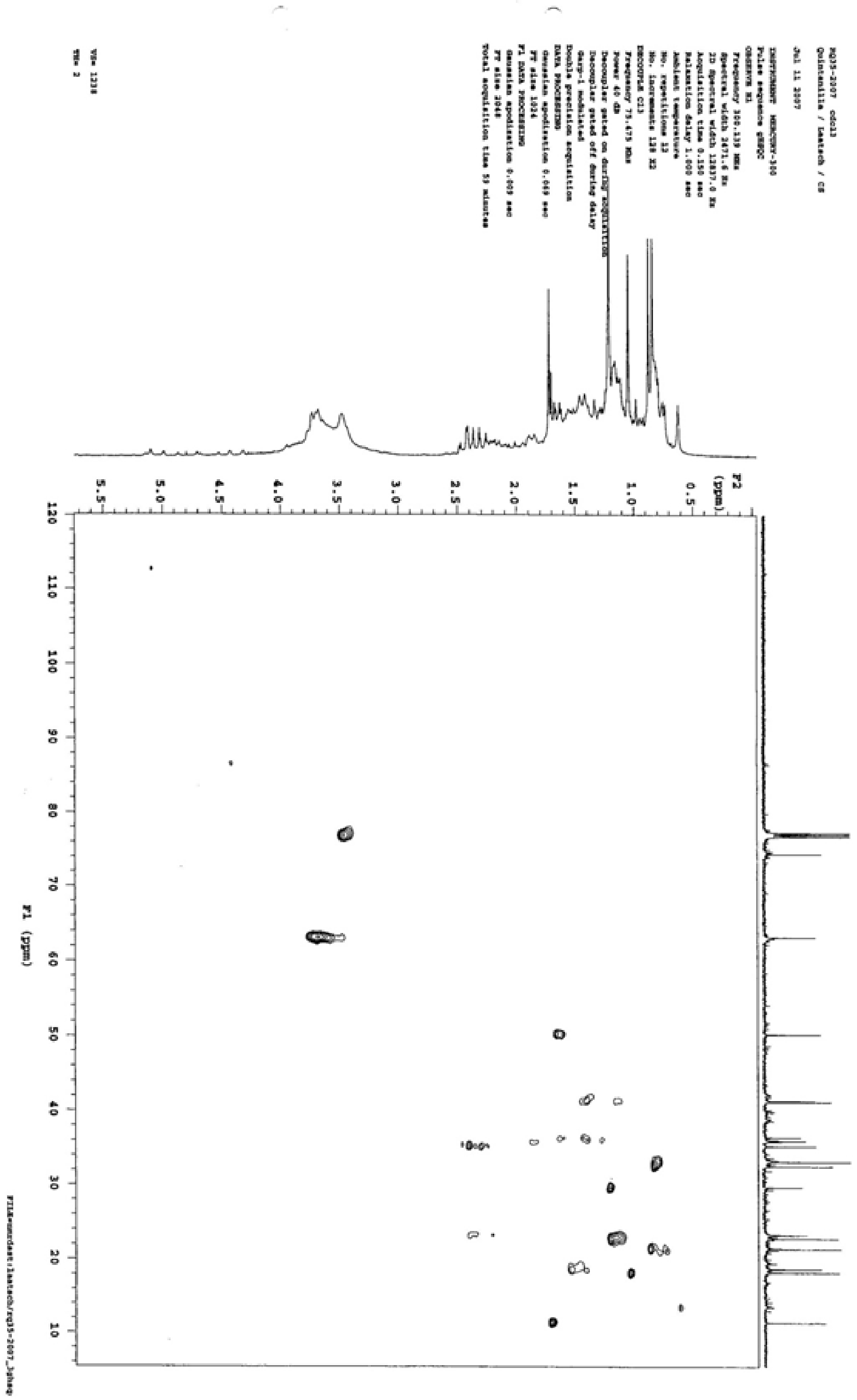

S20: HMBC spectrum of compound **3** ($CDCl_3$, 300 MHz).

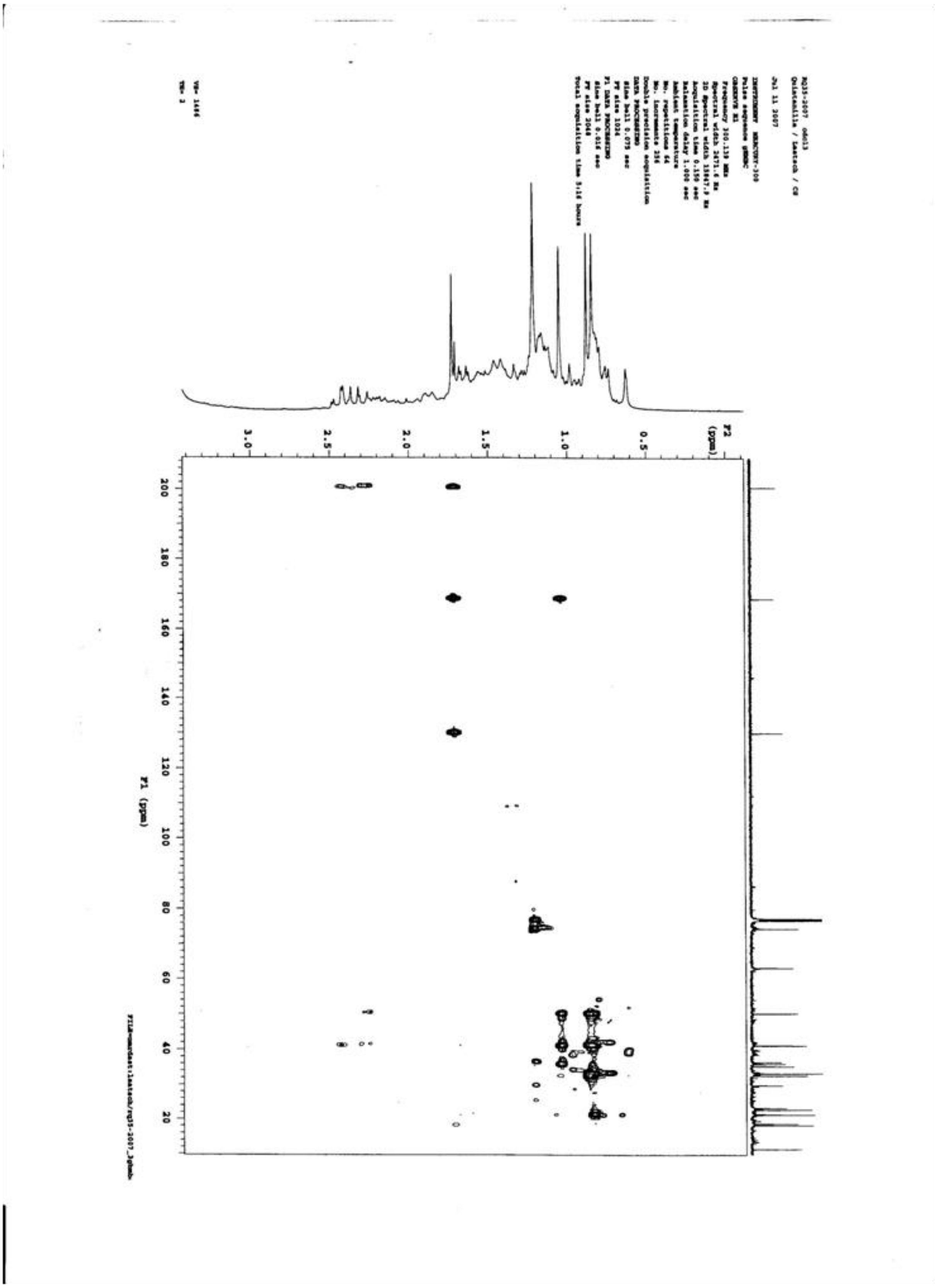

S21: ^{1}H-NMR spectrum of compound **4** (D_6-DMSO, 300 MHz).

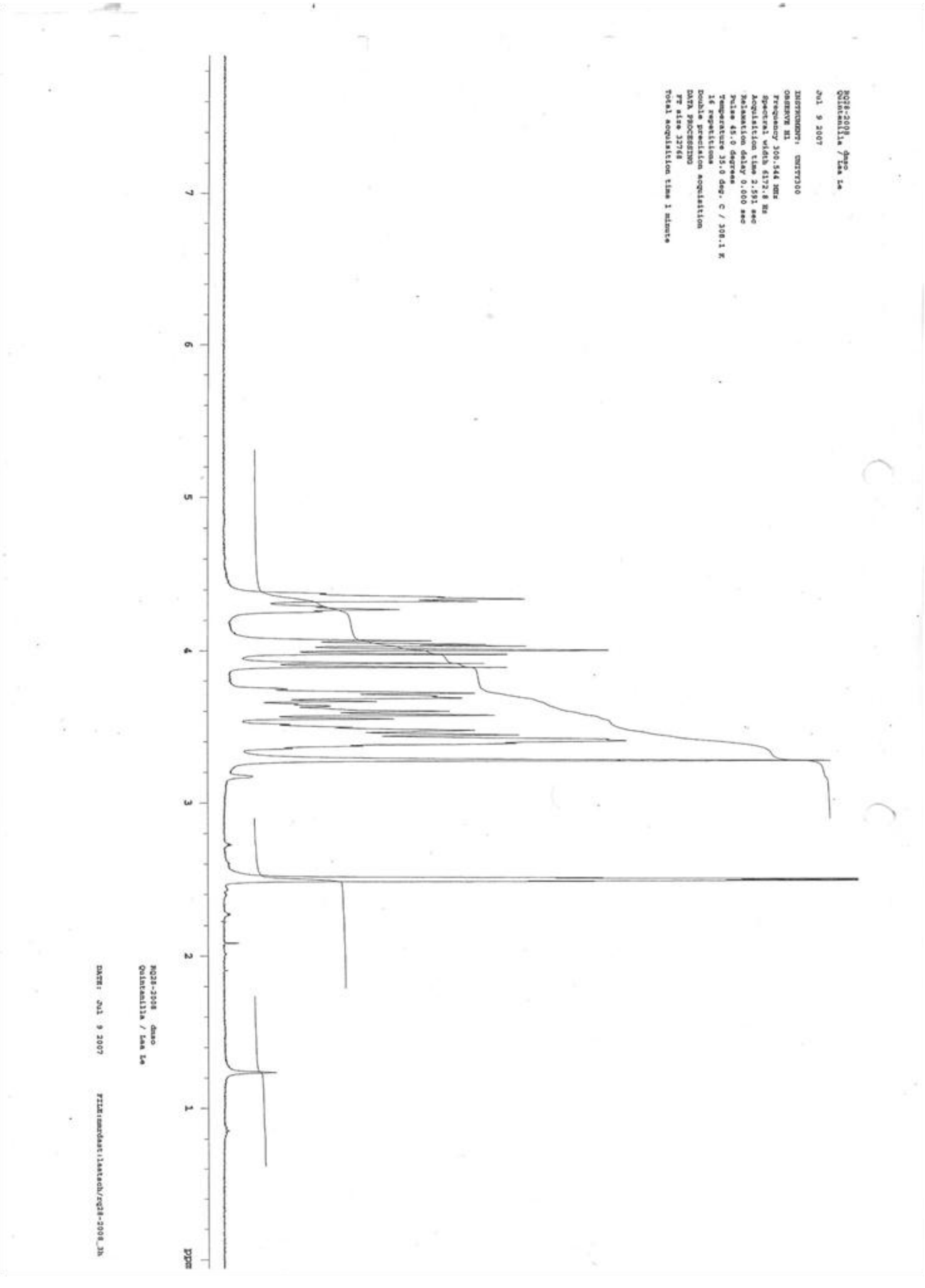

S22: ^{13}C-NMR spectrum of compound **4** (D_6-DMSO, 125 MHz).

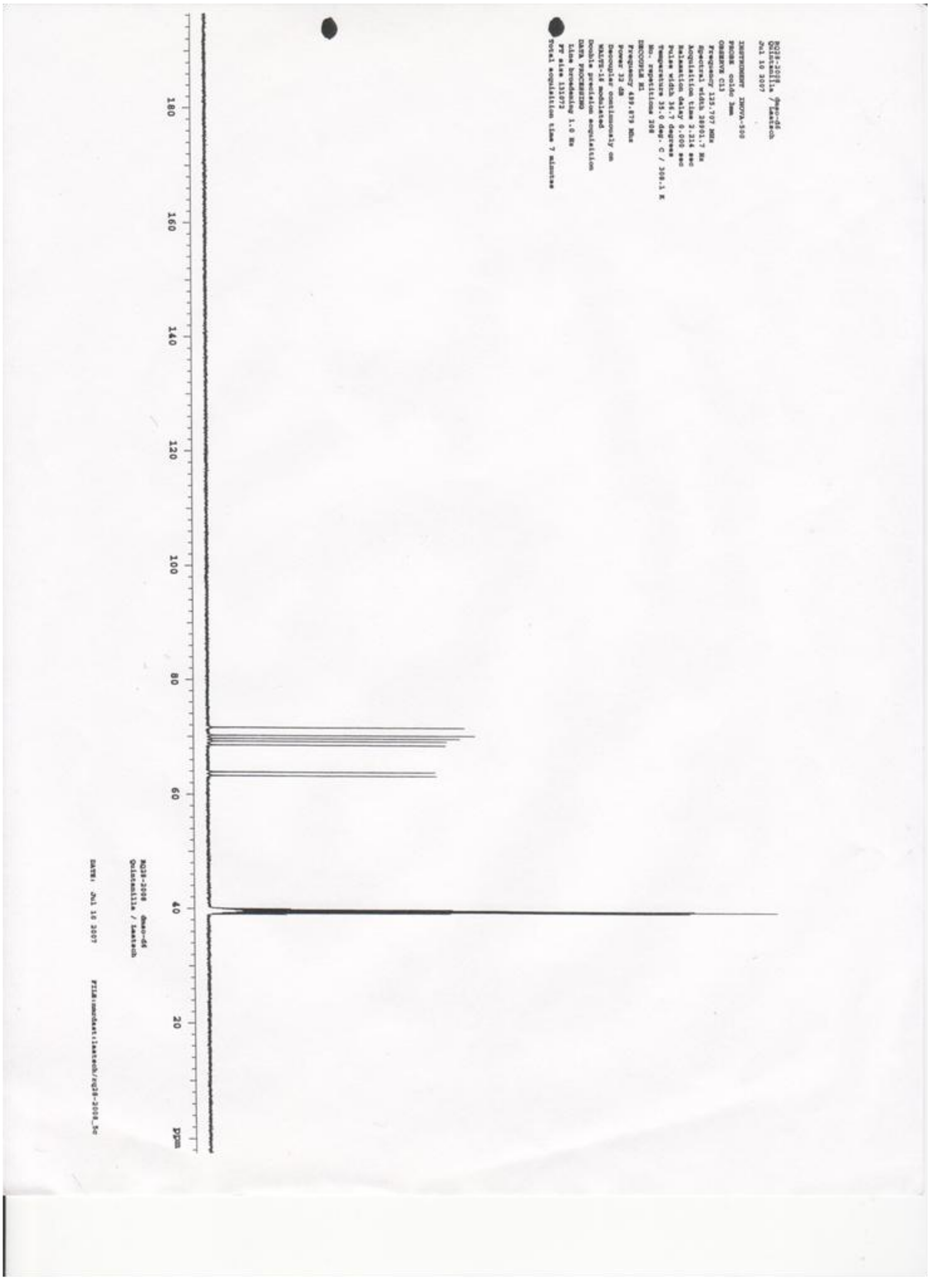

S23: DEPT spectrum of compound **4** (D_6-DMSO, 125 MHz).

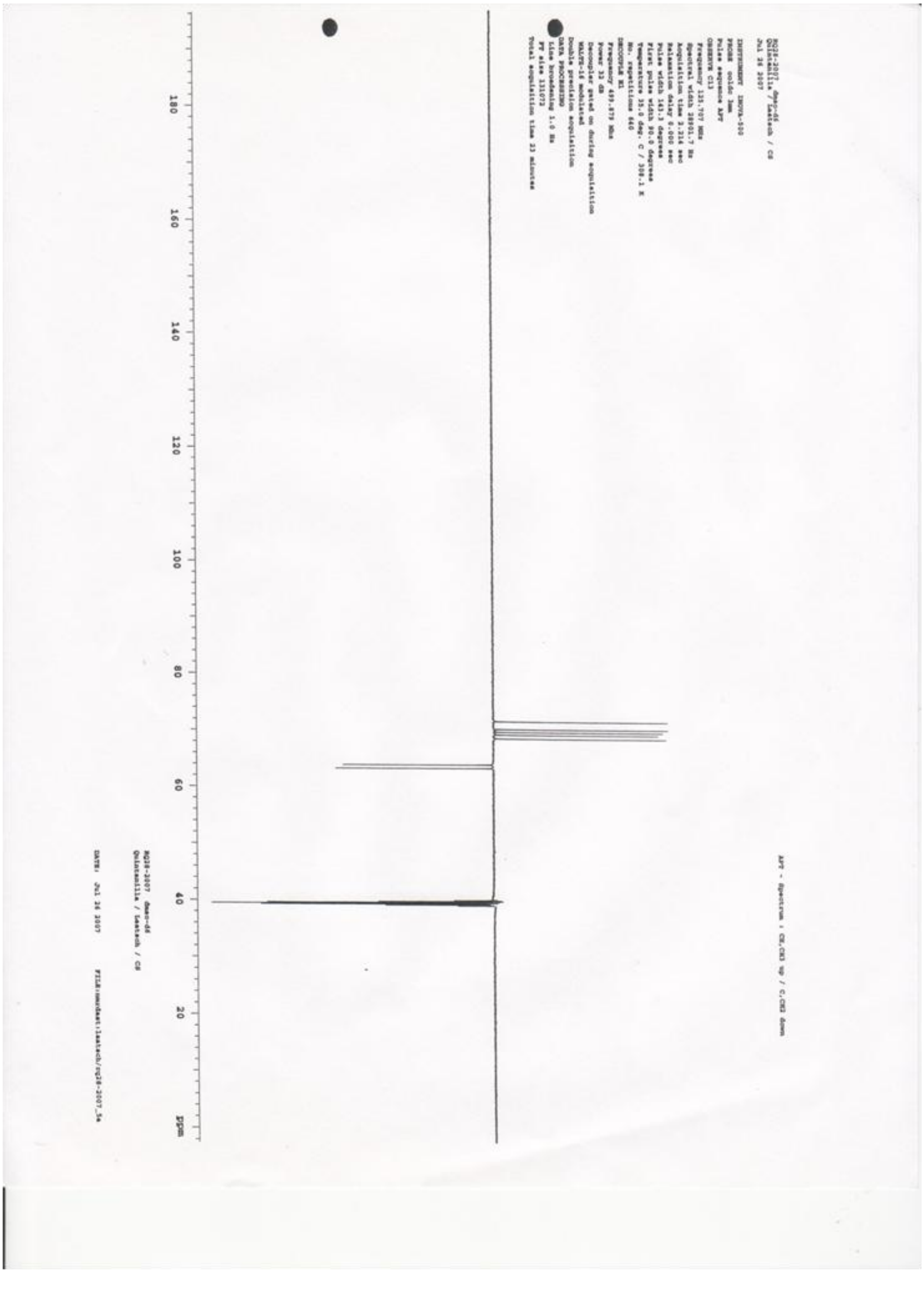

S24: COSY spectrum of compound **4** (D_6-DMSO, 300 MHz).

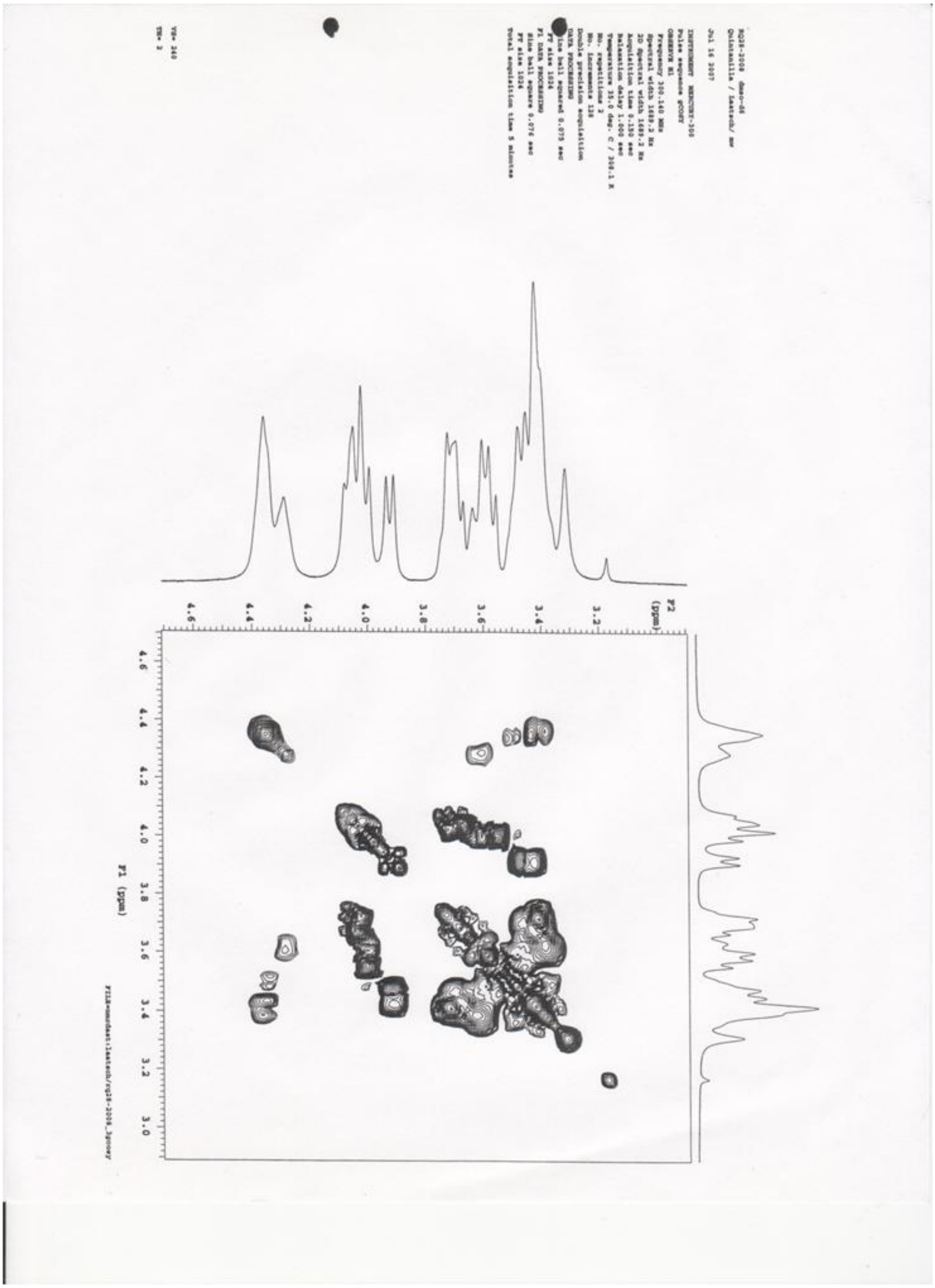

S25: HSQC spectrum of compound **4** (D_6-DMSO, 300 MHz).

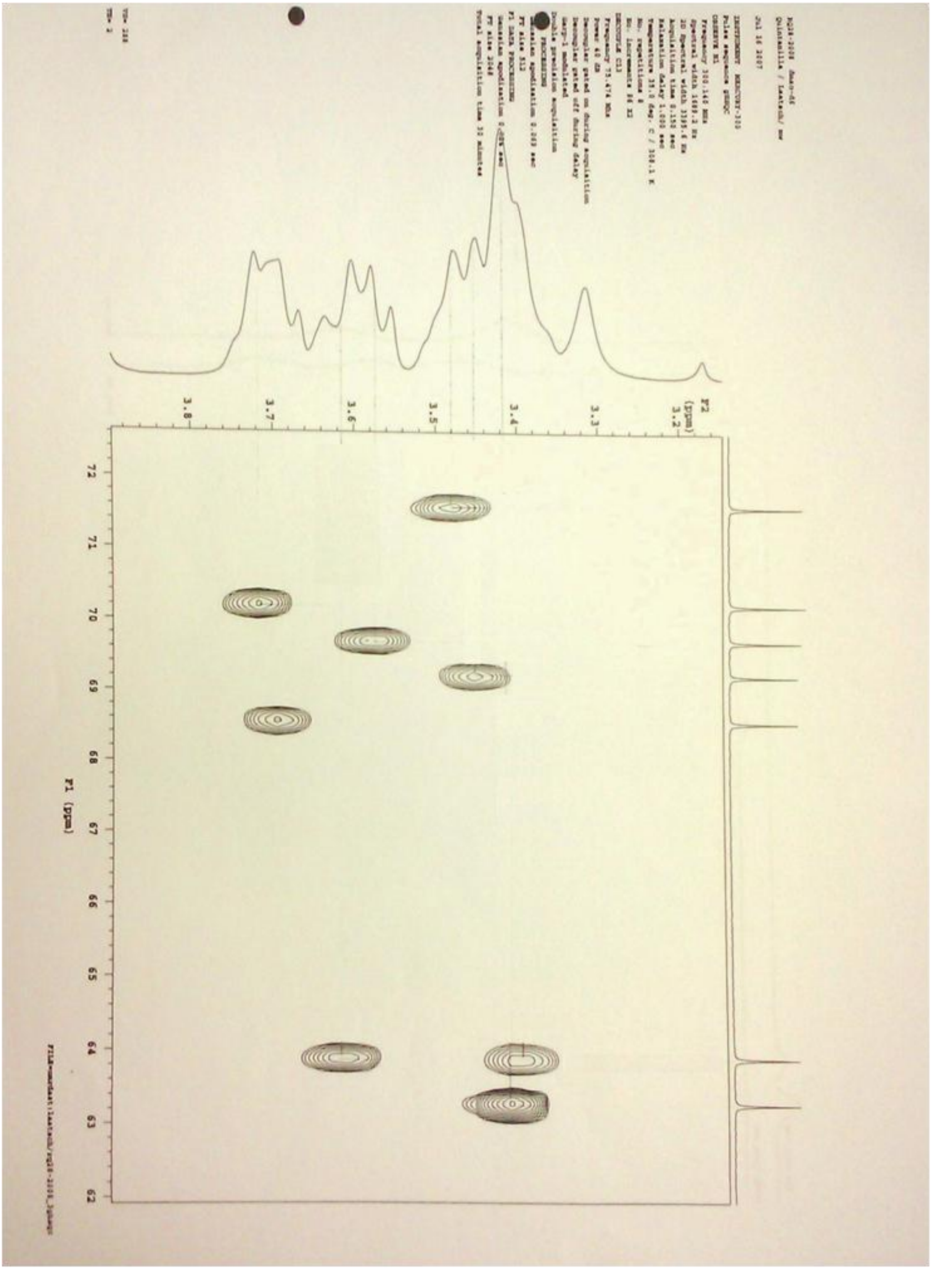

S26: ^{1}H-NMR spectrum of compound **4** after deuterium exchange (D_6-DMSO, 300 MHz).

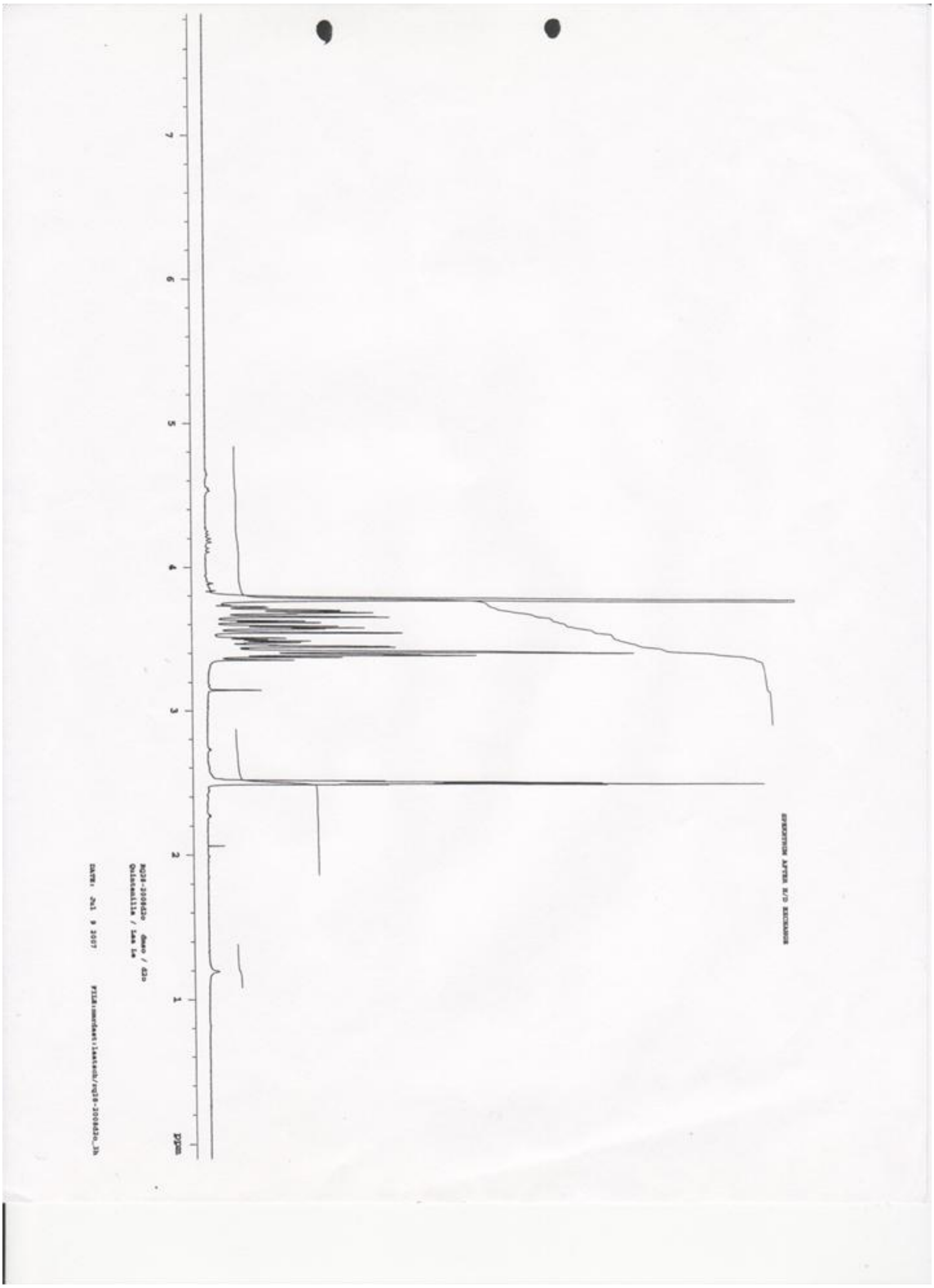

S27: Physico-chemical data of Compounds **1**, **2** and **4**.

Hentriacontane (**1**): White solid; R_f = 0.94 (hexane–$CHCl_3$, 1:1); m. p. 67 ℃, 68 ℃; IR (ATR): ν_{max} = 2956, 2916, 2848, 1413, 1463, 1378, 730, 720 cm^{-1}; EI-MS (70 eV): *m/z* (%) = 436 $[M]^+$ (10), 323 (5), 253 (6), 183 (10), 141 (12), 113 (17), 99 (25), 85 (59), 71 (79), 57 (100), 43 (50).

*(+)-13*S,*14*R,*15-Trihydroxy*-ent-*labd-7-ene* (**2**). Colorless oil; R_f = 0.25 ($CHCl_3$–EtOAc, 1:1); $[\alpha]_D^{20}$: +2 (c 4.22, $CHCl_3$); IR (ATR): ν_{max} = 3370, 2915, 1640, 1462, 1372, 1018, 720 cm^{-1}; DCI-MS (NH_3): *m/z* = 342 $[M + NH_4]^+$; EI-MS (70 eV): *m/z* (%) = 324 $[M]^+$ (5), 306 (1), 245 (10), 204 (100), 161 (12), 135 (11), 121 (23), 109 (26), 95 (15), 69 (7), 55 (2).

D-Glycero-D-galacto-heptitol (**4**). White solid; m. p. 185.6 ℃; $[\alpha]_D^{20}$: +14 (c 0.4, in 5% aq. ammoniumheptamolybdate); +72 (c ca. 0.4 in acidified 5% aq. ammoniumheptamolybdate); the foregoing solution was diluted with 25% 1N H_2SO_4, according to Richtmeyer *et al*. (Richtmeyer, N.K.; Hudson, C.S. The rotation of polyols in ammonium molybdate solutions. *J. Am. Chem. Soc*. **1951** *73*, 2249–2250); IR (ATR): ν_{max} 3240, 2925, 1410, 1305, 1210, 1100, 1005, 890, 630 cm^{-1}; The ^1H- and ^{13}C-NMR spectra in DMSO-d_6 were identical with those in the literature; ESI-MS (+)-mode: *m/z* = 235 $[M + Na]^+$, (-)-mode: *m/z* = 211 $[M - H]^-$; (+)-ESI-HR-MS: *m/z* 235.07886 $[M + Na]^+$ (calcd. for $C_7H_{16}O_7Na$: 235.07882).

S28: ^1H-(400 MHz) and ^{13}C-NMR (100 MHz) data of compound **1** ($CDCl_3$).

	^{13}C		^1H
Position	δ	(DEPT)	δ, mult, (*J* in Hz)
1, 31	14.1	CH_3	0.89 t (6.8)
2, 30	22.7	CH_2	1.27 m
3, 29	31.9	CH_2	
4, 28	29.4	CH_2	
5-27	29.7	CH_2	

S29: ^{1}H-(400 MHz) and ^{13}C-NMR (100 MHz) data of compound **2** ($CDCl_3$).

	^{13}C		^{1}H
Position	δ	(DEPT)	δ, mult, (*J* in Hz)
1	39.2	CH_2	0.94 m, 1.79 br d (11)
2	18.8	CH_2	1.44 m, 1.51 m
3	42.2	CH_2	1.14 m, 1.41 m
4	32.9	C_q	-
5	50.1	CH	1.16 dd (12, 4.9)
6	23.7	CH_2	1.87 m, 1.97 m
7	122.3	CH	5.37 br s
8	135.0	C_q	-
9	55.2	CH	1.54 m
10	36.9	C_q	-
11	20.7	CH_2	1.26 m, 1.43 m
12	40.7	CH_2	1.31 m, 1.85 m
13	75.0	C_q	-
14	76.2	CH	3.48 dd (5.7, 3.65)
15	63.1	CH_2	3.74 d (5.7)
16	23.3	CH_3	1.22 s
17	22.2	CH_3	1.68 s
18	33.1	CH_3	0.84 s
19	21.8	CH_3	0.86 s
20	13.5	CH_3	0.75 s
OH			3.60 br s

S30: ^{1}H-(400 MHz) and ^{13}C-NMR (100 MHz) data of compound **2** (DMSO-d_6).

Position	^{13}C δ	^{13}C (DEPT)	^{1}H δ, mult, (*J* in Hz)
1	38.6	CH_2	0.91 m, 1.86 m
2	18.4	CH_2	1.40 m, 1.47 m
3	42.0	CH_2	1.17 m, 1.72 m
4	32.7	C_q	-
5	49.8	CH	1.11 m
6	23.3	CH_2	-
7	121.3	CH	5.32 s
8	135.6	C_q	-
9	55.0	CH	1.46 m
10	36.6	C_q	-
11	20.0	CH_2	-
12	41.9	CH_2	1.13 m, 1.37 m
13	73.1	C_q	-
14	76.0	CH	3.28 m
15	62.6	CH_2	3.31 m, 3.60 d (8)
16	22.3	CH_3	0.97 s
17	22.1	CH_3	1.64 s
18	33.0	CH_3	0.83 s
19	21.7	CH_3	0.85 s
20	13.5	CH_3	0.72 s
OH			4.09 br s, 4.46 br m

S31: ^{1}H-(300 MHz) and ^{13}C-NMR (125 MHz) data of compound **4** (DMSO-d_6).

Position	^{13}C δ	^{13}C (DEPT)	^{1}H δ, mult, (*J* in Hz)
1	63.9	CH_2	3.39 m, 3.61 m
2	71.6	CH	3.48 m
3	68.6	CH	3.69 m
4	69.2	CH	3.45 m
5	69.7	CH	3.57 m
6	70.2	CH	3.72 m
7	63.3	CH_2	3.42 m
OH			3.92 d, 3.99 m, 4.02 m, 4.07 m, 4.29 t, 4.34 m, 4.35 m
